# Regulation of H-type angiogenesis and permeability in the subchondral bone of osteoarthritis: the role of Slit3 and the Robo4/Rac1-GTP/ROS axis

**DOI:** 10.3389/fcell.2026.1787805

**Published:** 2026-05-01

**Authors:** Xiaolei Chen, Xiaoxin He, Xue Lin, Jiangbo Yan, Di Xue, Lufei Shao, Gangning Feng, Xin Zhao, Long Ma, Kuanmin Tian, Hui Wang, Zhibin Lan, Zhidong Lu, Peng Li, Qunhua Jin

**Affiliations:** 1 The Third Ward of Orthopaedic Department, General Hospital of Ningxia Medical University, Yinchuan, China; 2 The Spinal orthopaedic Department, General Hospital of Ningxia Medical University, Yinchuan, China; 3 First Clinical Medical School, Ningxia Medical University, Yinchuan, China; 4 Institute of Osteoarthropathy, Institute of Medical Sciences, General Hospital of Ningxia Medical University, Yinchuan, China; 5 Ningxia Key Laboratory of Clinical and Pathogenic Microbiology, General Hospital of Ningxia Medical University, Yinchuan, China; 6 The Neurology Department, General Hospital of Ningxia Medical University, Yinchuan, China

**Keywords:** angiogenesis, osteoarthritis (OA), Rac1-GTP, Robo4, Slit3, subchondral bone, vascular permeability

## Abstract

Subchondral bone H-type angiogenesis and increased permeability are critical in osteoarthritis (OA) progression, yet the underlying regulatory mechanisms remain unclear. This study utilized proteomic analysis to identify significantly elevated expression of Slit guidance ligand 3 (Slit3) and Ras-related C3 botulinum toxin substrate 1 (Rac1) in the subchondral bone of patients with OA, both associated with angiogenesis and permeability. Experimental validation revealed that Slit3 and Rac1-GTP, rather than total Rac1, drive increased H-type angiogenesis and permeability. To further investigate, we exogenously added Slit3 recombinant protein to mimic the effect of non-ECs secreting Slit3 in the subchondral bone microenvironment on endothelial cells (ECs). Exogenous Slit3 significantly promoted migration, tube formation, and permeability in H-type ECs. An anti-Roundabout guidance receptor 4 (Robo4) antibody inhibited these effects and suppressed Rac1-GTP expression and reactive oxygen species (ROS) levels in ECs. Further investigation revealed that Rac1-GTP and ROS inhibitors could block the effects of exogenous Slit3 on H-type endothelial cell migration, tube formation, and permeability. In in vivo experiments, knockout of Slit3 aggravated early OA in destabilization of the medial meniscus (DMM) mice by altering cartilage and subchondral bone structure but alleviated late-stage OA. Moreover, Slit3 promoted H-type angiogenesis and permeability in the subchondral bone of DMM mice through the Robo4/Rac1-GTP signaling pathway, consistent with *in vitro* findings. Collectively, this study demonstrates that Slit3 mediates increased H-type angiogenesis and permeability in OA subchondral bone via the Robo4/Rac1-GTP/ROS signaling axis. Modulating Slit3 may offer stage-specific therapeutic strategies for OA.

## 1 Introduction

Osteoarthritis (OA) is the most prevalent age-related degenerative joint disease worldwide, often causing joint pain, impaired mobility, deformities, and, in severe cases, disability (Barnett, 2018; Martel-Pelletier et al., 2016). During OA progression, the formation of new H-type blood vessels in the subchondral bone, characterized by high expression of cluster of differentiation 31 (CD31) and endomucin (EMCN) (CD31^hi^EMCN^hi^), is a key feature (Peng et al., 2020; Kusumbe et al., 2014). These vessels breach the microcracks in the subchondral bone plate, penetrating the avascular cartilage and acting as a bridge between the cartilage and subchondral bone (Mapp and Walsh, 2012; Walsh et al., 2010). As H-type angiogenesis and vascular permeability increase, inflammatory factors from the subchondral bone and blood vessels infiltrate the cartilage, thereby inducing cartilage degeneration and accelerating OA progression (Zhu et al., 2020; Lu et al., 2018). However, the exact mechanisms underlying the formation of H-type blood vessels and the associated increase in permeability remain unclear. Emerging evidence suggests that molecular communication between osteoblasts, which regulate bone remodeling, and H-type endothelial cells (H-type ECs), a specific subtype of endothelial cells characterized by high CD31 and Endomucin expression (CD31^hi^Emcn^hi^) in the subchondral bone, is critical in establishing “angiogenesis–osteogenesis coupling.” Osteoblast-secreted factors, including vascular endothelial growth factor (VEGF) and Slit3, promote endothelial cell proliferation, vessel assembly, and stabilization, thereby influencing the generation and permeability of H-type blood vessels (Xu et al., 2018; Ramasamy et al., 2014; Kim et al., 2018; Gerber et al., 1999).

Slit3, a member of the Slit guidance ligand (SLIT) family, was initially discovered as an axon guidance molecule in the nervous system (Long et al., 2004). However, recent studies have shown that Slit3 is expressed in non-neuronal tissues and is involved in various physiological processes, including angiogenesis, stem cell regulation, and cancer progression (Paul et al., 2013; Geutskens et al., 2012; Basha et al., 2023). Slit3, secreted by osteoblasts and other cell types, acts as a potent pro-angiogenic factor, increasing the number of H-type ECs. Furthermore, Slit3 has been shown to promote wound healing associated with H-type vessels, and in fracture models, Slit3 has been identified as a key regulator of H-type angiogenesis, thereby aiding bone fracture repair (Xu et al., 2018). However, the role and underlying mechanisms of Slit3 in the generation and permeability of H-type blood vessels in OA subchondral bone remain unclear. Studies have found that Robo family proteins, members of the immunoglobulin superfamily of cell adhesion molecules, serve as the primary receptors for Slit ligands (Howitt et al., 2004; Legg et al., 2008). Binding between Robo receptors and Slit3 is required for various physiological and pathological functions, including angiogenesis, inflammatory cell chemotaxis, tumor cell migration, and fibrosis (Basha et al., 2023; Zhang et al., 2009; Jiang et al., 2019). To date, four Robo receptors (Robo1–4) have been identified in mammals, with Robo4 being recognized as the endothelial cell-specific receptor within the Robo family (Dickson and Gilestro, 2006). When Slit3 is co-incubated with anti-Robo4 antibodies, it completely inhibits Slit3-induced migration of HUMECs (Zhang et al., 2009). Additionally, mesenchymal stem cell-derived Slit3 can stimulate Robo4-positive ECs to form vascular networks and guide vascular development (Paul et al., 2013). However, the role and mechanisms of the Slit3-Robo4 pathway in H-type angiogenesis remain unreported.

Beyond molecular communication between different cell types influencing angiogenesis and permeability, the actin cytoskeleton, endothelial cell proliferation, migration, differentiation, and the integrity of intercellular junctions are crucial in regulating vascular function (Carmeliet, 2003; Claesson-Welsh et al., 2021; Eelen et al., 2020). Rac1, a member of the Rho GTPase family, regulates cytoskeletal dynamics, migration, proliferation, and cell differentiation (Arnold et al., 2017; Ridley, 2001). It also mediates endothelial cell signaling induced by angiogenic factors such as VEGF. Rac1 continuously switches between an active GTP-bound state and an inactive GDP-bound state, with guanine nucleotide exchange factors (GEFs) promoting the conversion of Rac1-GDP to the active Rac1-GTP form, thereby facilitating its regulatory role in vascular physiology (Keller-Pinter et al., 2017). Rac1-GTP influences cytoskeletal changes by regulating intercellular junction molecules such as Zonula Occludens-1 (ZO-1) and cadherins, which directly impacts angiogenesis and permeability (Wojciak-Stothard and Ridley, 2002). However, the role of Rac1-GTP in regulating the generation and permeability of H-type blood vessels in OA subchondral bone remains unknown.

In this study, we demonstrated that non-endothelial cell–derived Slit3 in the subchondral bone plays an important role in regulating H-type angiogenesis and vascular permeability, thereby promoting the progression of osteoarthritis (OA). This finding helps further improve the understanding of the pathogenesis of osteoarthritis and provides a potential new molecular target for its treatment.

## 2 Materials and methods

### 2.1 Patients and samples

The human tibial plateau samples were collected from 30 patients diagnosed with knee osteoarthritis (KOA) who underwent total knee arthroplasty (TKA) at the First Affiliated Hospital of Ningxia Medical University between 25 September 2022, and 25 December 2022. The patients were aged between 60 and 70 years. The subchondral bone was categorized into damaged area (OA sample) and corre sponding undamaged area (control sample). OA diagnosis was based on the American College of Rheumatology’s diagnostic criteria, and patients with secondary OA due to trauma or connective tissue diseases were excluded (Altman et al., 1986).

### 2.2 Endothelial cell isolation and culture

Femurs and tibias were harvested from wild-type C57BL/6 mice (age: 6 weeks; weight: 15 g) and immediately placed in sterile phosphate-buffered saline (PBS) lacking Ca^2+^ and Mg^2+^. Bone tissues were thoroughly ground using a sterile mortar and pestle, followed by enzymatic digestion with collagenase (Sigma) to generate a single-cell suspension. Endothelial cells (ECs) were isolated by magnetic-activated cell sorting (MACS) using CD31-specific antibodies. The purified ECs were seeded onto culture dishes pre-coated with fibronectin and maintained in endothelial basal medium (EBM-2) supplemented with EGM-2 SingleQuots (Lonza). The final culture medium contained 2% FBS, 0.1% VEGF, 0.04% hydrocortisone, 0.4% hFGF-B, 0.1% R3-IGF-1, 0.1% ascorbic acid, 0.1% hEGF, 0.1% GA-1000, and 0.1% heparin. During the first passage, CD31-based MACS was repeated to ensure purity, and cells were replated for continued culture. Culture medium was refreshed every 3 days, and cells were passaged upon reaching confluence. All cells were maintained in a humidified incubator at 37 °C with 5% CO_2_. Endothelial cells between passages 2 and 5 were used for downstream experiments.

### 2.3 Primary chondrocytes isolation and culture

Mouse cartilage tissue was washed three times with phosphate-buffered saline (PBS) under sterile conditions and cut into small pieces of approximately 0.5 mm^3^. The cartilage pieces were then incubated with three volumes of trypsin (Gibco, 25300–054) at 37 °C for 30 min. After digestion, the mixture was centrifuged at 40 *g* for 5 min to remove the supernatant, and the cartilage pieces were resuspended in sterile PBS and centrifuged again to wash once more. Next, the cartilage pieces were digested with five volumes of 0.2% type II collagenase (Gibco, 17101–015) in a 37 °C orbital shaker for 16 h. The resulting digest was centrifuged at 40 *g* for 5 min to separate the supernatant from the undigested tissue. The undigested tissue was then subjected to a second digestion with five volumes of 0.2% type II collagenase for an additional 16 h to maximize cell yield. Cells from the combined digests were collected by centrifugation at 300 *g* for 5 min and resuspended in MEK/F12 medium containing 10% fetal bovine serum (Gibco, 10099-141). Cells were cultured in a humidified incubator at 37 °C with 5% CO_2_, and the medium was changed every 3 days. When the cells reached approximately 80% confluence, they could be passaged or used for downstream experiment.

### 2.4 Construction of OA mouse model

**FIGURE 1:**
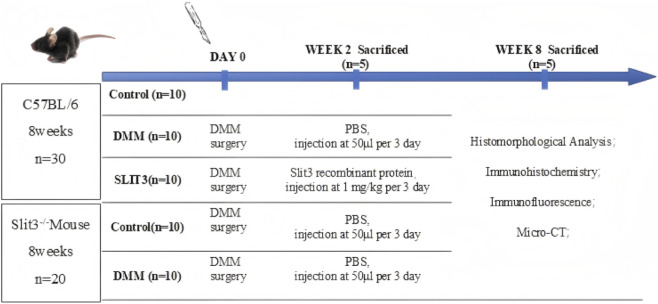
Animal experiment flowchart.

Thirty healthy adult female wild-type C57BL/6 mice (age: 8 weeks; weight: 10–25 g) were purchased from the Laboratory Animal Center of Ningxia Medical University, China. Twenty female Slit3^−/−^ mice (age: 8 weeks; weight: 20–25 g) were purchased from Jiangsu Saiye Biotechnology Co., Ltd. Complete gene knockout was constructed using CRISPR/Cas technology to generate F0 generation positive mice. F0 generation mice were bred with wild-type mice to obtain F1 heterozygotes, which were subsequently bred to produce F2 homozygotes. All mice were housed in a specific pathogen-free environment under standard conditions (22 °C ± 1 °C, 55% humidity, 12/12-h light/dark cycle) with free access to food and water. We established an OA model in mice by performing right knee medial meniscus destabilization (DMM). Briefly, after intraperitoneal injection of pentobarbital (40 mg/kg) for anesthesia, the skin and joint capsule of the right knee were incised, and the anterior cruciate ligament of the right knee was transected to release the anterior horn of the meniscus. In the control group, only the skin and joint capsule were incised, and the meniscal ligament was left intact (He et al., 2024). Wild-type mice were randomly divided into three groups (10 mice per group): control (sham surgery), OA (DMM), and Slit3 recombinant protein intervention (DMM + SLIT3) groups. Slit3^−/−^ mice were divided into two groups (10 mice per group): the control (sham surgery) and OA (DMM) groups. Mice in the DMM + SLIT3 group received intraperitoneal injection of Slit3 recombinant protein (1 mg/kg) twice weekly, while the remaining mice were injected with an equal volume of PBS following the same schedule. Mice were euthanized by cervical dislocation after being anesthetized with intraperitoneal pentobarbital (40 mg/kg) at 2- and 8-weeks post-surgery, and knee joint tissues were collected for subsequent experiments. The animal experimental procedure is shown in Figure 1.

### 2.5 Western blot analyses

ECs were seeded at a density of 5 × 10^5^ cells per well in a 6-well plate and cultured for 24 h at 37 °C under 5% CO_2_. After incubation, cells were washed three times with PBS on ice. Then, 80 µL of mammalian protein extraction reagent (cat. No. 78501; Thermo Fisher Scientific, USA) was added per well. After cell lysis and centrifugation, the total protein extract was obtained, and protein concentration was measured using the BCA assay. Next, 20 µg of protein from each sample was separated on a 10% or 15% SDS-PAGE gel and transferred to a PVDF membrane, which was blocked with 5% skim milk at room temperature for 1 h. The membrane was then incubated overnight at 4 °C with the specific primary antibody. After five washes with TBST (0.05% Tween-20), the membrane was incubated with the secondary antibody at room temperature for 1 h. Protein bands were visualized using an enhanced chemiluminescence (ECL) kit (cat. No. RM0021; ABclonal, China), and images were captured using the ChemiDoc™ imaging system (Bio-Rad Laboratories, Inc.). Protein expression levels were quantified using ImageJ v1.8.0 software (National Institutes of Health). The following primary antibodies were used: Anti-Slit guidance ligand 3 (Slit3, 1:1000; cat. No. PA5-104142; Invitrogen), roundabout guidance receptor 4 (Robo4, 1:1000; cat. No. DF14939; Affinity), ras-related C3 botulinum toxin substrate 1 (Rac1, 1:1000; cat. No. 66122-1-IG; Proteintech), Rac1-GTP (1:1000; cat. No. 26903; Neweast), intercellular adhesion molecule 1 (ICAM1, 1:1000; cat. No. 10020-1-AP; Proteintech), cluster of differentiation 31 (CD31, 1:1500; cat. No. 28083-1-AP; Proteintech), endomucin (EMCN, 1:1000; cat. No. 67854-1-IG; Proteintech), Matrix Metalloproteinase 13 (MMP13, 1:1000; cat. No. 18165-1-AP; Proteintech), Collagen type II alpha 1 chain (Col2a1,1:1000; cat. No. 28459-1-AP; Proteintech), Glyceraldehyde-3-phosphate dehydrogenase (GAPDH; 1:5000; cat. No. 60004-1-Ig; Proteintech) and β-actin (1:5000; cat. No. 66009-1-Ig; Proteintech). The secondary antibodies used were horseradish peroxidase-conjugated goat anti-rabbit (1:10,000; cat. No. ab205718; Abcam) and anti-mouse IgG (1:10,000; cat. No. ab205719; Abcam).

### 2.6 Cell viability assays

In brief, cell viability of ECs was measured using the Cell Counting Kit-8 (CCK-8) (cat. No. CK04; Dojindo, Japan). ECs were seeded at a density of 5000 cells per well in a 96-well plate. The cells were then treated with IL-1β (5, 10, 50, 100, 200, 500 ng/mL) and the Rac1-GTP inhibitor NSC23766 (10, 50, 100, 200, 500 µM) for 24 h at 37 °C with 5% CO_2_. After removing the culture medium, 100 µL of 10% CCK-8 solution was added to each well, followed with incubation at 37 °C for 1 h. Finally, cell viability was measured by detecting the absorbance at 460 nm using a microplate reader (Infinite® 200 PRO; Tecan, Switzerland).

### 2.7 Wound healing assay

ECs were seeded uniformly at a density of 5 × 10^5^ cells per well in a 6-well plate and cultured for 12 h at 37 °C with 5% CO_2_ until the cell confluence reached approximately 70%. A scratch was then made on the EC monolayer, and the cells were incubated in serum-free medium at 37 °C with 5% CO_2_ for 24 h. Images were captured at 0h, 4h, 12h, and 24 h using a fluorescence microscope (cat. No. BX53; Olympus Corporation, Japan).

### 2.8 ECs migration, invasion, and tube formation assay

For the ECs migration assay, cells were seeded at a density of 2.5 × 10^5^ cells per well in the upper chamber of a transwell insert and cultured in serum-free medium. The lower chamber contained 700 µL of complete medium with 10% FBS. Cells were incubated at 37 °C with 5% CO_2_ for 24 h. After incubation, the transwell inserts were placed into a 12-well plate with 1 mL of 4% paraformaldehyde per well for fixation. Then, 1 mL of 0.1% crystal violet-ammonium oxalate staining solution was added to each well and incubated at room temperature (25 °C) for 20 min. After staining, the inserts were washed, air-dried, and images were captured using a fluorescence microscope (cat. No. BX53; Olympus Corporation, Japan). The number of migrated cells was counted. For the ECs invasion assay, Matrix-Gel (standard type) was mixed with phenol-free H-DMEM medium at a ratio of 1:8, and 60 µL of the mixture was added to the upper chamber of the transwell insert. The mixture was incubated at 37 °C for 3 h to allow gelation, and unbound matrix gel was then aspirated. Afterward, 100 µL of serum-free medium was added to the lower chamber and incubated at 37 °C for 30 min to confirm the absence of fluid leakage. ECs were then seeded in the upper chamber, and the subsequent steps were the same as for the migration assay. For the ECs tube formation assay, ECs were cultured for 24 h in medium containing 0.2% FBS at 37 °C with 5% CO_2_. Matrix-Gel (standard type) was mixed with phenol-free H-DMEM at a ratio of 2:1 to create a 66.7% matrix gel solution. Then, 60 µL of the matrix gel solution was added to each well of a 96-well plate and incubated at 37 °C for 1 h to solidify. ECs were seeded at a density of 5 × 10^4^ cells per well and cultured at 37 °C with 5% CO_2_ for 24 h. Images were captured using a fluorescence microscope (cat. No. BX53; Olympus Corporation, Japan) and the tube formation was quantified.

### 2.9 ECs permeability assay

ECs were seeded at a density of 2.5 × 10^5^ cells per well in the upper chamber of a transwell insert. The lower chamber was filled with 700 µL of phenol-free H-DMEM medium, and the cells were cultured at 37 °C with 5% CO_2_ for 24 h. After washing, FITC-Dextran (MW 10,000) was mixed with 500 µL of phenol-free H-DMEM medium and added to the upper chamber of the transwell insert. At designated time points (0h, 0.5h, 1h, 4h, 16h, 24h, 36h, 48 h), 100 µL of medium was aspirated from the lower chamber and transferred into a 96-well plate. Absorbance was measured at a wavelength of 485 nm using a microplate reader (Infinite® 200 PRO; Tecan, Switzerland).

### 2.10 Fluorescence analyses

After culturing ECs at 37 °C with 5% CO_2_ for 24 h, they were washed with PBS. The cells were then fixed with 4% paraformaldehyde at room temperature (25 °C) for 10 min, followed by permeabilization with 0.2% Triton-100 for 5 min. After washing with PBS, the cells were blocked with 1% BSA for 30 min. For immunofluorescence analysis, ECs were incubated overnight at 4 °C with the primary antibody. After washing with TBST, the cells were incubated with the secondary antibody at room temperature (25 °C) for 2 h, protected from light. For rhodamine-conjugated phalloidin staining, ECs were incubated with 200 µL of rhodamine-conjugated phalloidin working solution (100 nM; Beijing Solarbio Science & Technology, China) at room temperature (25 °C) for 30 min, protected from light. For ROS analysis, ECs were incubated with 200 µL of CM-H2DCFDA working solution (5 µM; Beyotime, China) at room temperature (25 °C) for 40 min, protected from light. After washing with PBS, the cells were stained with DAPI (5 µM; Invitrogen, USA) for nuclear staining at room temperature (25 °C) before mounting. Images were captured using a fluorescence microscope (cat. no. BX53; Olympus Corporation, Japan) and quantified using ImageJ v1.8.0 (National Institutes of Health). To ensure consistency, all images were captured using the same settings. The primary antibodies used were as follows: Anti-CD31 (1:300; cat. No. 28083-1-AP; Proteintech), EMCN (1:300; cat. No. 67854-1-IG; Proteintech), VE-cadherin (1:400; cat. no. 2500T; Cell Signaling Technology), ZO-1 (1:500; cat. no. ab96587; Abcam), connexin 43 (1:200; cat. no. ab235282; Abcam).

### 2.11 Histology and immunohistochemical staining

The knee joint tissues were fixed in 4% paraformaldehyde at 4 °C for 24 h and then decalcified in 10% EDTA at room temperature (25 °C) for 4 weeks. After dehydration with graded ethanol, the tissues were embedded in paraffin and sectioned to a thickness of 4 μm for subsequent experiments. For hematoxylin and eosin (H&E) staining, following the instructions of the H&E staining kit (cat. no. G1076; Servicebio, China), the sections were dewaxed and dehydrated at 20 °C, followed by hematoxylin staining and eosin staining. According to the Safranin O-fast green staining kit (cat. no. G1053; Servicebio, China), the sections were pre-treated similarly to H&E staining. The sections were stained with fast green at 20 °C for 6 min, washed, dehydrated, and then stained with Safranin O for 3 min. Masson’s trichrome staining was performed according to the Masson’s staining kit instructions (cat. no. G1006; Servicebio, China). For immunohistochemistry (IHC), the same method was used to prepare the knee joint tissue sections. After dewaxing and rehydration, antigen retrieval was performed with 0.1% trypsin at 37 °C for 30 min. Then, endogenous peroxidase activity was quenched by incubating with 3% hydrogen peroxide at 25 °C for 10 min. The sections were blocked with 5% goat serum (cat. no. G1208; Servicebio, China) at 37 °C for 30 min. The sections were incubated overnight at 4 °C with the following primary antibodies: Anti-Slit3 (1:500; cat. No. PA5-104142; Invitrogen), Robo4 (1:300; cat. No. DF14939; Affinity), Rac1 (1:300; cat. No. 66122-1-IG; Proteintech), Rac1-GTP (1:300; cat. No. 26903; Neweast), ICAM1 (1:500; cat. No. 10831-1-AP; Proteintech), CD31 (1:500; cat. No. 11265-1-AP; Proteintech). After washing, the sections were incubated with the following secondary antibodies at room temperature (25 °C) for 30 min: horseradish peroxidase-conjugated goat anti-rabbit (1:10,000; cat. no. ab205718; Abcam) and anti-mouse IgG (1:10,000; cat. no. ab205719; Abcam). Finally, the sections were stained with DAB (cat. no. G1212; Servicebio, China) and counterstained with hematoxylin at 25 °C for 5 min. Images were captured using a light microscope (cat. no. CX43; Olympus Corporation, Japan) and analyzed with ImageJ v1.8.0 software (National Institutes of Health).

### 2.12 Micro-CT analysis

After carefully removing the skin and muscles surrounding the right knee joint of the mice, the tissue was fixed in 4% paraformaldehyde at room temperature for 48 h. Scanning was performed using a micro-CT scanner (SkyScan 1176; Bruker Belgium S.A./N.V.) with a resolution of 9 µm/pixel. The analyzed parameters included bone volume fraction (BV/TV), trabecular separation (Tb.Sp), trabecular thickness (Tb.Th), and trabecular number (Tb.N).

### 2.13 Liquid chromatography-tandem mass spectrometry and label-free quantification of tissue proteomes

LC-MS/MS analysis was performed using a Q Exactive mass spectrometer and an Easy nLC (Thermo Fisher Scientific, Inc.) mass spectrometer in positive ion mode. The nitrogen temperature was set to 180 °C with a flow rate of 3 L/min. Parallel Reaction Monitoring (PRM) transitions were evaluated within a scan range of 300–1,800. Data were collected using LC-MS/MS and then subjected to a database search (uniprot.org) for protein identification. The criteria for differential expression of proteins (DEPs) were |logFC| > 1 and a p-value <0.05. Functional annotation of DEPs was performed using Gene Ontology (GO; https://www.geneontology.org) and Kyoto Encyclopedia of Genes and Genomes (KEGG; http://geneontology.org/) pathway enrichment analysis. Results were plotted using R (version 4.3.3) software (https://www.r-project.org). Visualization of the differential expression proteins was done using the “pheatmap” and “ggplot2” packages in R. A heatmap was generated with “pheatmap,” and a volcano plot was created using “ggplot2.”

### 2.14 Data and statistical analyses

All data are expressed as the mean ± SD. All independent experiments were repeated at least three times. All statistical analyses were performed using GraphPad Prism 9.0 (GraphPad Software, Inc.). For comparisons between two groups, an unpaired Student’s t-test was used. For comparisons of ≥3 groups, homogeneity of variance was first tested, followed by one-way ANOVA and Tukey’s multiple comparisons post-test. A p-value <0.05 was considered statistically significant.

## 3 Results

### 3.1 Quantitative proteomics and validation

This study enrolled 30 patients diagnosed with osteoarthritis (OA) who underwent total knee arthroplasty (TKA). We initially assessed their weight-bearing anteroposterior X-ray images, magnetic resonance imaging (MRI) scans, and intraoperative joint cartilage appearance and measured the cartilage thickness (Supplementary Figure S1A). The patients were subsequently evaluated using the OARSI score, Visual Analog Scale (VAS) score, and Kellgren-Lawrence (K-L) grading system. The results revealed that all 30 patients were classified as K-L grade 4, with an average OARSI score of 14.87 ± 1.042 and an average VAS score of 6.367 ± 0.9643 (Supplementary Figure S1B). Cartilage thickness measurements at the medial femoral condyle (MF) were 0.6 ± 0.51 mm, at the medial tibial plateau (MT) 0.63 ± 0.54 mm, at the lateral femoral condyle (LF) 2.17 ± 0.6 mm, and at the lateral tibial plateau (LT) 2.44 ± 0.48 mm. Notably, the cartilage thickness in the OA region was significantly reduced compared to the control region (Supplementary Figure S1C). The subchondral bone thickness of the medial tibial plateau (MT) was 6.36 ± 2.01 mm, while that of the lateral tibial plateau (LT) was 2.3 ± 1.3 mm (Supplementary Figure S1D). These findings confirm that the clinical samples exhibit typical OA characteristics and are suitable for subsequent experimental analysis.

Proteomic data analysis revealed 82 differentially expressed proteins between the control and OA groups, with 42 proteins downregulated and 40 proteins upregulated. Notably, the expression levels of SLIT3, Rac1, and VEGF were significantly elevated in the OA group (Supplementary Figures S1A, B). Functional enrichment analysis of these differentially expressed genes (DEGs) revealed that Gene Ontology (GO) analysis was primarily enriched in leukocyte migration, chemotaxis, and adhesion processes, while Kyoto Encyclopedia of Genes and Genomes (KEGG) pathway analysis indicated that these DEGs were mainly associated with cell proliferation and adhesion (Supplementary Figures S1C, D). These findings suggest that SlLIT3, Rac1, and VEGF may be involved in OA progression and are potentially linked to the regulation of angiogenesis and vascular permeability.

### 3.2 SLIT3 and Rac1-GTP are potential contributors to increased H-type vascular growth and permeability in subchondral bone of OA

**FIGURE 2:**
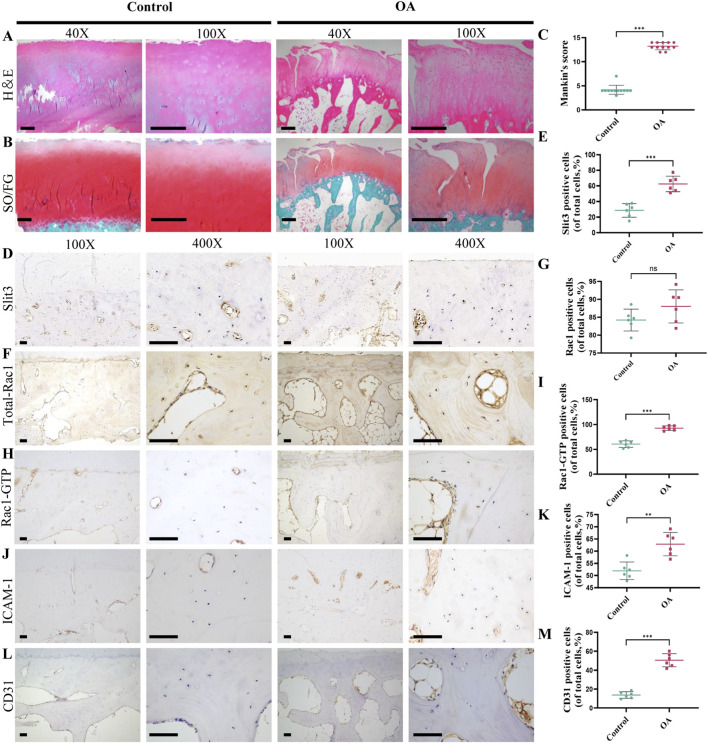
Slit3, Rac1-GTP, and vascular function are closely related to OA. **(A)** H&E staining results of clinical samples. Scale bars, left, 500 μm; right, 200 μm. **(B)** Safranin-O/fast green staining results of clinical samples. Scale bars, left, 500 μm; right, 200 μm. **(C)** Mankin’s scores of articular cartilage pathology. n = 12. **(D)** Representative immunohistochemical staining images of slit3 in subchondral bone. Scale bars, left, 200 μm; right, 50 μm. **(E)** Proportion of slit3-positive cells. n = 6. **(F)** Representative immunohistochemical staining images of Total-Rac1 in subchondral bone. Scale bars, left, 200 μm; right, 50 μm. **(G)** Proportion of Total-Rac1-positive cells. n = 6. **(H)** Representative immunohistochemical staining images of Rac1-GTP in subchondral bone. Scale bars, left, 200 μm; right, 50 μm. **(I)** Proportion of Rac1-GTP-positive cells. n = 6. **(J)** Representative immunohistochemical staining images of ICAM1 in subchondral bone. Scale bars, left, 200 μm; right, 50 μm. **(K)** Proportion of ICAM1-positive cells. n = 6. **(L)** Representative immunohistochemical staining images of CD31 in subchondral bone. Scale bars, left, 200 μm; right, 50 μm. **(M)** Proportion of CD31-positive cells. n = 6. All quantitative data are expressed as mean ± standard deviation, *P < 0.05; **P < 0.01; ***P < 0.001. Comparisons between two groups were performed using Student’s t-test.

To validate the proteomic findings, we conducted further experimental analyses on clinical samples. Hematoxylin and eosin (H&E) staining and Safranin O-fast green staining revealed more pronounced cartilage damage and higher Mankin’s scores in the OA group compared to the Control group (Figures 2A–C). To investigate whether Slit3 and Rac1 are involved in the pathological changes of subchondral bone in OA, we performed immunohistochemistry to assess the expression of SLIT3, Total-Rac1, and Rac1-GTP in the subchondral bone. The results demonstrated significantly elevated levels of SLIT3 and Rac1-GTP in the OA group, with no significant differences in Total-Rac1 expression between the two groups (Figures 2D–I). To further explore changes in angiogenesis and vascular permeability during OA progression, we examined key factors involved in these processes, namely, ICAM-1 and CD31. The findings showed that both ICAM-1 and CD31 were significantly upregulated in the OA group (Figures 2J–M). These results indicate that H-type angiogenesis and vascular permeability are significantly increased in the subchondral bone of OA, with corresponding upregulation of Slit3 and Rac1-GTP expression.

**FIGURE 3:**
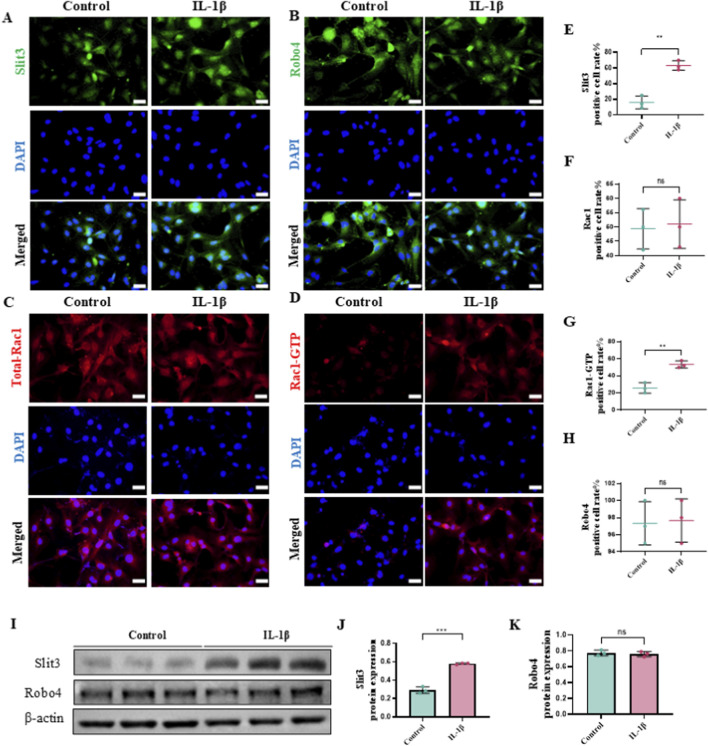
Inflammatory stimulation increases the expression of slit3 and Rac1-GTP in ECs, while no changes are observed in robo4 and Total-Rac1. IL-1β (100 ng/mL) was used to treat ECs for 24 h. **(A)** Representative immunofluorescence staining images of slit3, **(B)** robo4, **(C)** Total-Rac1, and **(D)** Rac1-GTP. Scale bar, 50 μm. **(E)** Quantification of slit3, **(F)** robo4, **(G)** Total-Rac1, and **(H)** Rac1-GTP positive cell proportions by immunofluorescence. n = 3. **(I)** Western blot analysis of slit3 and robo4. **(J,K)** Quantification of protein expression levels of slit3 and robo4. n = 3. All quantitative data are expressed as mean ± standard deviation, *P < 0.05; **P < 0.01; ***P < 0.001. Comparisons between two groups were performed using Student’s t-test.

To further confirm whether Slit3 and Rac1-GTP are involved in the changes in angiogenesis and permeability in OA, we simulated OA-induced endothelial cell inflammation by treating ECs with IL-1β and examined the expression of relevant proteins. First, we determined the optimal drug concentrations for IL-1β and the Rac1-GTP inhibitor NSC23766 using the CCK-8 assay, which revealed the optimal concentrations to be 100 ng/mL for IL-1β and 100 μM for NSC23766 (Supplementary Figures SA, B). Immunofluorescence staining showed that Slit3 expression was minimal in control ECs but significantly increased following IL-1β treatment (Figures 3A,E). The expression of Slit3’s receptor, Robo4, did not show significant changes in IL-1β-treated ECs (Figures 3B,H). Moreover, while Total-Rac1 expression remained unchanged in IL-1β-treated ECs compared to controls, Rac1-GTP expression was significantly elevated (Figures 3C,D,F,G). Western blot analysis confirmed these findings, consistent with the immunofluorescence results (Figures 3I–K). These observations suggest that in response to OA-related inflammatory stimuli, Slit3, which is normally expressed at very low levels in ECs, shows a slight increase, while its receptor Robo4 remains unaffected by inflammation. Importantly, Rac1-GTP, rather than Total-Rac1, appears to be involved in OA progression in ECs. Collectively, we conclude that Slit3 and Rac1-GTP are potential contributors to the increased H-type angiogenesis and vascular permeability in the subchondral bone of OA.

### 3.3 Exogenous slit3 promotes migration, tube formation, and permeability of H-type ECs

**FIGURE 4:**
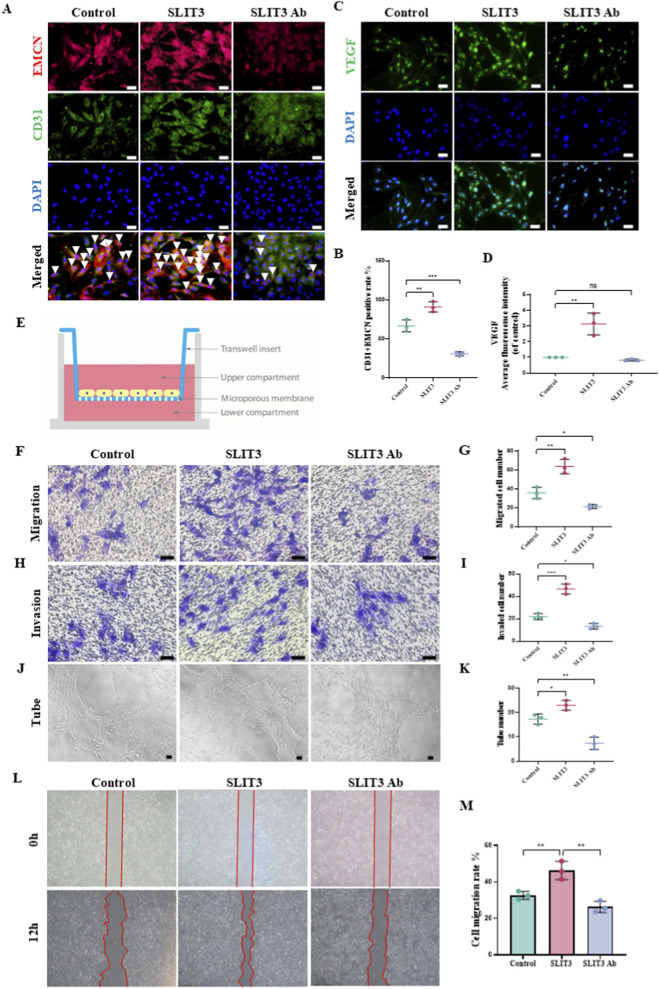
Exogenous addition of slit3 promotes the migration, invasion, and tube formation ability of H-type vascular ECs. ECs were co-incubated with exogenous recombinant slit3 protein (1.25 μg/mL) or slit3 neutralizing antibody (1.25 μg/mL) for 24 h. **(A)** Representative immunofluorescence images of H-type vascular markers CD31 and EMCN co-staining. Scale bar, 50 μm. **(B)** Quantification of CD31+EMCN positive cell proportion by immunofluorescence. n = 3. **(C)** Representative immunofluorescence staining images of VEGF. Scale bar, 50 μm. **(D)** Quantification of average VEGF fluorescence intensity. n = 3. **(E)** Schematic of the Transwell assay. **(F)** Representative crystal violet-oxalate staining images of ECs migrated through a 3 μm pore membrane. Scale bar, 100 μm. **(G)** Quantification of migrated ECs. n = 3. **(H)** Representative crystal violet-oxalate staining images of ECs invading through a matrix-gel-covered 3 μm pore membrane. Scale bar, 100 μm. **(I)** Quantification of invaded ECs. n = 3. **(J)** Representative images of EC tube formation on matrix-gel. Scale bar, 50 μm. **(K)** Quantification of tube numbers per field. n = 3. **(L)** Representative images taken at 0h, and 12 h after scratch formation on a monolayer of ECs. Scale bar, 100 μm. **(M)** Quantification of cell migration area. n = 3. All quantitative data are presented as mean ± standard deviation, *P < 0.05; **P < 0.01; ***P < 0.001. Comparisons between two groups were performed using Student’s t-test.

To assess the role of Slit3 in modulating the function of H-type vascular ECs in the subchondral bone microenvironment, we externally added recombinant Slit3 protein to ECs to simulate the effects of Slit3 secreted by non-ECs in the OA subchondral bone microenvironment. Immunofluorescence staining revealed that, with increasing concentrations of external Slit3, both the fluorescence intensity and the number of Slit3-positive ECs increased. Based on these observations, we selected a concentration of 1.25 μg/mL of recombinant Slit3 protein for subsequent experiments (Supplementary Figures S4A–C). Meanwhile, we determined the optimal concentration of the Slit3 neutralizing antibody (1.25 μg/mL) for further experiments based on immunofluorescence analysis and cell viability assays (Supplementary Figures S5A–C). To confirm whether ECs exhibit H-type vascular EC characteristics (CD31^hi^ and EMCN^hi^), we performed immunofluorescence staining for CD31 and EMCN, two key markers of these cells. Our results showed high expression of both markers in ECS cells, with Slit3 addition increasing the number of CD31^hi^ and EMCN^hi^ positive cells. However, when Slit3-neutralizing antibodies were used, the number of CD31^hi^ and EMCN^hi^ positive cells significantly decreased (Figures 4A,B). We then assessed the effects of Slit3 on H-type angiogenesis and permeability. VEGF, a potent pro-angiogenic factor, is essential for both promoting angiogenesis and maintaining vascular stability. Immunofluorescence analysis showed that external addition of Slit3 upregulated VEGF expression, while Slit3 inhibition resulted in the opposite effect (Figures 4C,D). ZO-1, Connexin 43, and VE-cadherin are key adhesion molecules involved in intercellular junctions in ECs. Our staining results indicated that Slit3 treatment led to a significant reduction in fluorescence intensity for ZO-1 and Connexin 43 on the EC membrane and disrupted the continuity of VE-cadherin along endothelial cell junctions. In contrast, Slit3 inhibition produced the opposite effects (Supplementary Figures S6A–E). Additionally, the actin cytoskeleton plays a crucial role in regulating the vascular barrier. Slit3 treatment notably increased the formation of branched actin filaments within ECs, while Slit3 inhibition resulted in the opposite (Supplementary Figure S6F).

To further elucidate the impact of Slit3 on angiogenesis and permeability, we cultured ECs in transwell chambers to assess H-type vascular ECs’ migration, invasion, and tube formation abilities (Figure 4E). The results showed that external addition of Slit3 significantly increased the number of ECs that migrated through the transwell membrane pores and Matrigel matrix, as well as the number of tube-like structures formed by ECs on the Matrigel-coated surface. Conversely, Slit3 inhibition led to the opposite effects (Figures 4F–K). In addition, cell scratch assays demonstrated that external Slit3 promoted the migratory capacity of H-type ECs, while Slit3 inhibition had the opposite effect (Figures 4L,M). Collectively, these results confirm that exogenous Slit3 enhances the proliferation, migration, and invasion abilities of H-type vascular ECs, regulates adhesion molecules, and consequently promotes angiogenesis and increases vascular permeability.

### 3.4 Slit3 activates Rac1-GTP by binding to Robo4 and promotes migration, tube formation, and permeability of H-type ECs

**FIGURE 5:**
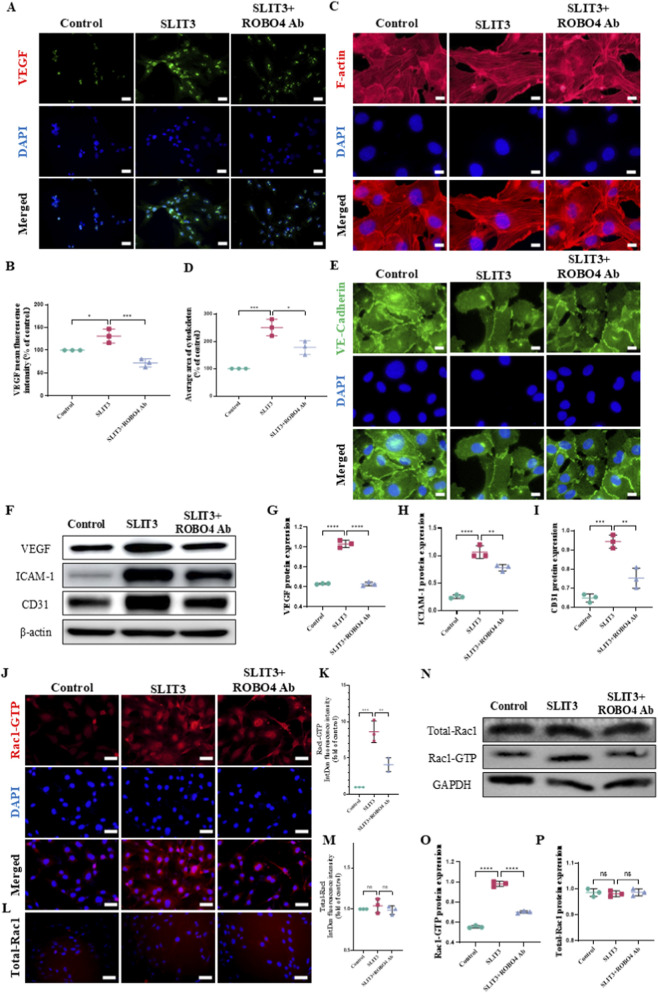
Robo4 neutralizing antibody inhibits slit3-induced increase in ECs permeability and Rac1-GTP activation. ECs were co-incubated with exogenous recombinant slit3 protein (1.25 μg/mL) alone or with Robo4 neutralizing antibody (1.25 μg/mL) for 24 h. **(A)** Representative immunofluorescence images of VEGF. Scale bar, 50 μm. **(B)** Quantification of average VEGF fluorescence intensity. n = 3. **(C)** Representative immunofluorescence images of ECs actin cytoskeleton. Scale bar, 20 μm. **(D)** Quantification of actin cytoskeleton immunofluorescence. n = 3. **(E)** Representative immunofluorescence images of VE-Cadherin. Scale bar, 20 μm. **(F)** Immunoblot analysis of VEGF, ICAM-1, and CD31. **(G–I)** Quantification of VEGF, ICAM-1, and CD31 protein expression. n = 3. **(J)** Representative immunofluorescence images of Rac1-GTP. Scale bar, 50 μm. **(K)** Quantification of Rac1-GTP immunofluorescence intensity. n = 3. **(L)** Representative immunofluorescence images of Total-Rac1. Scale bar, 50 μm. **(M)** Quantification of Total-Rac1 immunofluorescence intensity. n = 3. **(N)** Immunoblot analysis of Total-Rac1 and Rac1-GTP. **(O,P)** Quantification of Total-Rac1 and Rac1-GTP protein expression. n = 3. All quantitative data are presented as mean ± standard deviation, *P < 0.05; **P < 0.01; ***P < 0.001. Comparisons between two groups were performed using Student’s t-test.

To further elucidate the mechanism by which Slit3 influences H-type vascular formation and permeability, we utilized a Robo4 neutralizing antibody to inhibit the interaction between Slit3 and Robo4, and examined the subsequent effects on angiogenesis and permeability. Immunofluorescence staining revealed that the Robo4 neutralizing antibody effectively inhibited the Slit3-induced increase in VEGF fluorescence intensity and the formation of branching actin filaments in ECs (Figures 5A–D). Moreover, the Robo4 neutralizing antibody restored the continuity of VE-cadherin distribution along EC junctions, which Slit3 had disrupted (Figure 5E). Western blotting analysis showed that the Robo4 neutralizing antibody significantly suppressed the Slit3-induced upregulation of VEGF, ICAM-1, and CD31 expression (Figures 5F–I). Furthermore, we assessed the migration, invasion, and tube formation abilities of ECs and found that the Robo4 neutralizing antibody inhibited the Slit3-induced increase in ECs migrating through Transwell chamber pores and Matrigel matrix, as well as the formation of tube-like structures by ECs (Supplementary Figures S7A–F). These results confirm that blocking the interaction between Slit3 and Robo4 effectively suppresses the Slit3-induced increase in H-type angiogenesis and permeability.

We further discovered that inhibiting the interaction between Slit3 and Robo4 significantly suppressed the Slit3-induced increase in Rac1-GTP fluorescence intensity, while it had no effect on Total-Rac1 levels (Figures 5J–M), a finding that was corroborated by Western blotting analysis (Figures 5N–P). Additionally, we observed that the Robo4 neutralizing antibody effectively suppressed the Slit3-induced increase in ROS fluorescence intensity Supplementary Figures S7G, H). These results confirm that blocking the Slit3-Robo4 interaction can effectively inhibit the activation of Rac1-GTP and ROS induced by Slit3. Taken together, these experimental findings validate that Slit3 promotes H-type angiogenesis and permeability through its interaction with Robo4, and that the activation of Rac1-GTP and ROS plays a critical role in this process.

### 3.5 Slit3 promotes migration, tube formation, and permeability of H-type ECs via the Rac1-GTP/ROS pathway

**FIGURE 6:**
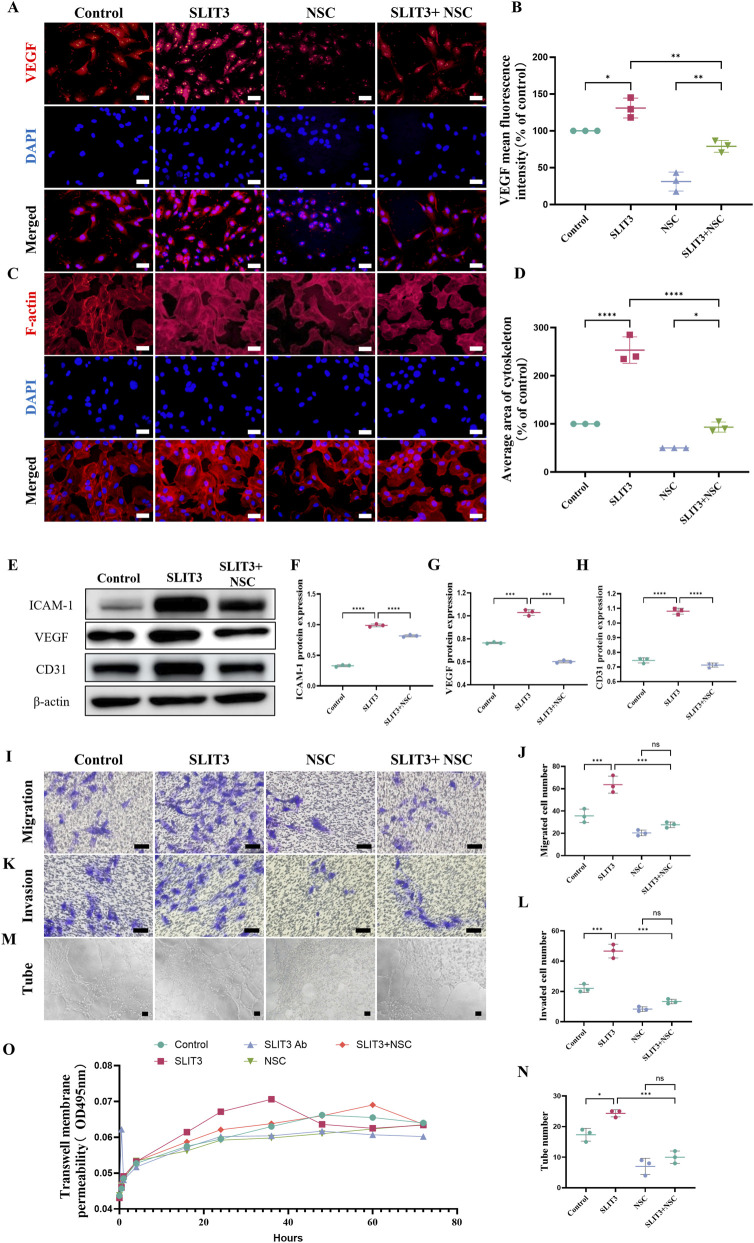
Rac1-GTP inhibitor NSC23766 inhibits slit3-induced ECs migration, invasion, and tube formation ability. Exogenous recombinant slit3 protein (1.25 μg/mL) and NSC23766 (50 μM) were co-incubated with ECs for 24 h separately or together. **(A)** Representative immunofluorescence images of VEGF. Scale bar, 50 μm. **(B)** Quantification of average VEGF fluorescence intensity. n = 3. **(C)** Representative immunofluorescence images of ECs actin cytoskeleton. Scale bar, 20 μm. **(D)** Quantification of actin cytoskeleton immunofluorescence. n = 3. **(E)** Immunoblot analysis of VEGF, ICAM-1, and CD31. **(F–H)** Quantification of VEGF, ICAM-1, and CD31 protein expression. n = 3. **(I)** Representative crystal violet-oxalate staining images of ECs migrating through a 3 μm pore membrane. Scale bar, 100 μm. **(J)** Quantification of the number of migrating ECs. n = 3. **(K)** Representative crystal violet-oxalate staining images of ECs invading through a 3 μm pore membrane covered with matrix gel. Scale bar, 100 μm. **(L)** Quantification of the number of invading ECs. n = 3. **(M)** Representative images of ECs tube formation on matrix gel. Scale bar, 50 μm. **(N)** Quantification of tube number per field. **(O)** Correlation analysis of ECs permeability and time variation. n = 3. All quantitative data are presented as mean ± standard deviation, *P < 0.05; **P < 0.01; ***P < 0.001. Comparisons between two groups were performed using Student’s t-test.

To further clarify the role of Rac1-GTP/ROS in Slit3-mediated regulation of H-type angiogenesis and permeability, we first employed the Rac1-GTP specific inhibitor NSC23766 to suppress the expression of Rac1-GTP in ECs. Immunofluorescence results showed that NSC23766 effectively inhibited the Slit3-induced increase in the number of CD31^hi^ + EMCN^hi^-positive cells (Supplementary Figures S8A, B). Additionally, inhibiting Rac1-GTP rescued the Slit3-induced increase in VEGF fluorescence intensity and the formation of branching actin filaments in ECs (Figures 6A–D). Western blotting analysis further confirmed that suppression of Rac1-GTP reversed the upregulation of VEGF, CD31, and ICAM1 protein expression (Figures 6E–H). We then assessed the migration, invasion, and tube formation abilities of ECs using Transwell assays. The results indicated that Rac1-GTP inhibition rescued the Slit3-induced increase in ECs’ penetration through Transwell pores and Matrigel matrix, as well as the number of tube-like structures formed by ECs (Figures 6I–N). Finally, we monitored the fluorescence intensity of FITC-DEXTRAN in the Transwell lower chamber to evaluate changes in permeability over time. In the first 4 h, neither Slit3 nor Rac1-GTP inhibition affected ECs’ permeability. However, between 16 and 36 h, Slit3 significantly increased EC permeability, while Slit3 and Rac1-GTP inhibition had the opposite effect. Furthermore, Rac1-GTP inhibition was able to rescue the Slit3-induced increase in permeability, consistent with our previous findings. After 48 h, neither Slit3 addition nor Slit3 and Rac1-GTP inhibition altered EC permeability (Figure 6O). These results suggest that inhibiting Rac1-GTP can rescue the Slit3-induced increase in H-type angiogenesis and permeability, and this effect is time-dependent.

Furthermore, we further investigated the role of ROS in Slit3-mediated regulation of H-type angiogenesis and permeability by using the ROS inhibitor Acetylcysteine (NAC). Immunofluorescence results revealed that inhibiting Rac1-GTP effectively rescued the Slit3-induced increase in ROS levels (Supplementary Figures S8A, B). We then observed the effects of NAC on vascular formation and permeability. NAC effectively suppressed the Slit3-induced increase in ROS in ECs (Supplementary Figures S9A, B). Moreover, immunofluorescence staining demonstrated that ROS inhibition could effectively reverse the Slit3-induced increase in VEGF fluorescence intensity and the formation of branching actin filaments in ECs (Supplementary Figures S9A–F). These findings indicate that inhibiting ROS can rescue the Slit3-induced increase in H-type angiogenesis and permeability. In conclusion, these experimental results suggest that Slit3 promotes H-type angiogenesis and permeability through the Rac1-GTP/ROS pathway.

### 3.6 Slit3 alleviates early OA progression in DMM mice but exacerbates late OA progression

**FIGURE 7:**
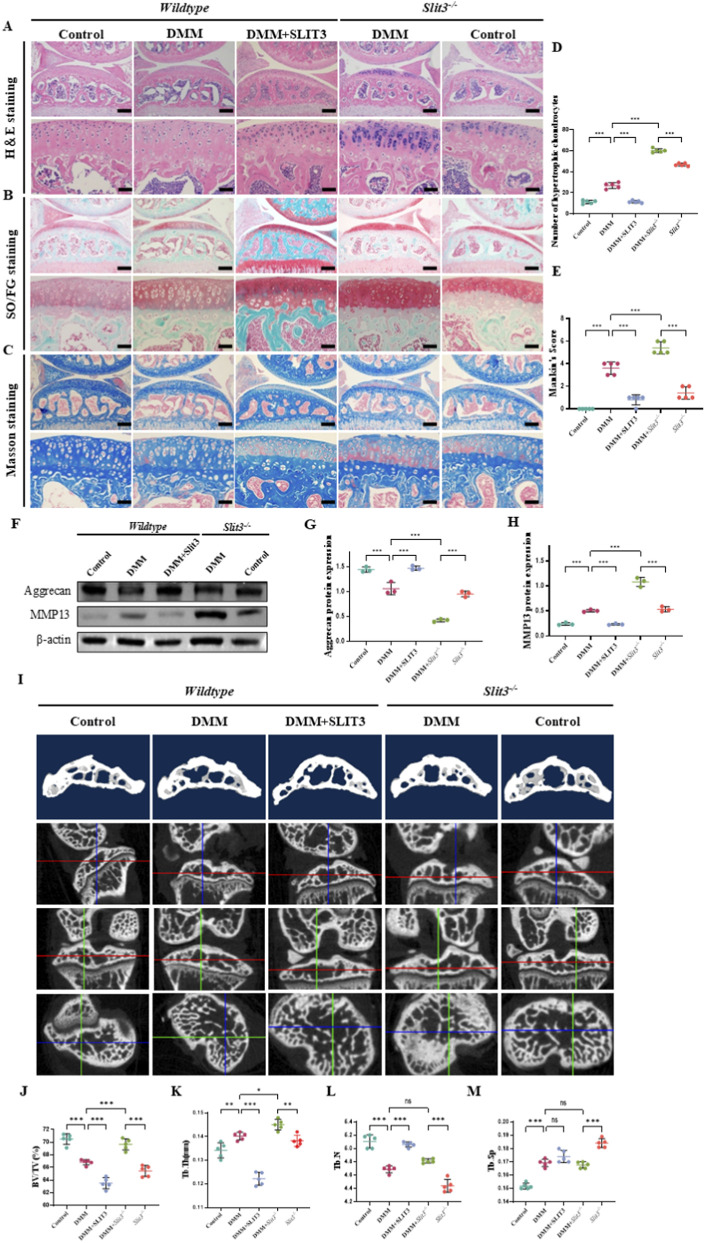
Knockout of Slit3 exacerbates early (2-week-old) OA in DMM mice by modulating the structure of cartilage and subchondral bone. **(A)** Representative H&E staining images of mouse knee joint tissues. Scale bar, 100 and 50 μm. **(B)** Representative Safranin O-fast green staining images of mouse knee joint tissues. Scale bar, 100 and 50 μm. **(C)** Representative Masson staining images of mouse knee joint tissues. Scale bar, 100 and 50 μm. **(D)** Quantification of hypertrophic chondrocyte number. n = 3. **(E)** Mankin’s score of joint cartilage pathological changes. n = 3. **(F)** Immunoblot analysis of Aggrecan and MMP13. **(G,H)** Quantification of Aggrecan and MMP13 protein expression. n = 3. **(I)** Representative 2D micro-CT scan and reconstructed images of tibial subchondral bone. Magnification ×10. Quantification of **(J)** bone volume fraction, **(K)** trabecular thickness, **(L)** trabecular number, and **(M)** trabecular separation based on micro-CT analysis. n = 3. All quantitative data are presented as mean ± standard deviation, *P < 0.05; **P < 0.01; ***P < 0.001. Comparisons between two groups were performed using Student’s t-test.

To further elucidate the regulatory role of Slit3 in osteoarthritis (OA) *in vivo*, we first modulated the expression of Slit3 in mice by intraperitoneal injection of recombinant Slit3 protein and by using Slit3^−/−^ mice. Immunohistochemistry results showed that, compared to the control group, the expression of Slit3 in the subchondral bone of wild-type DMM mice was upregulated. Additionally, intraperitoneal injection of recombinant Slit3 protein further increased the expression of Slit3 in DMM mice, whereas Slit3^−/−^ mice exhibited almost no detectable Slit3 expression (Supplementary Figures S10A, B). Subsequently, we assessed the cartilage condition of the 2-week group of mice using H&E, Safranin-O, and Masson’s staining. The results revealed that, in the wild-type control group, the joint cartilage was intact and smooth, with normal chondrocyte numbers, size, and structure. In contrast, wild-type DMM mice showed mild loss of cartilage smoothness and an increase in hypertrophic chondrocytes. Injection of Slit3 recombinant protein reduced the number of hypertrophic chondrocytes while increasing microcracks. However, Slit3^−/−^ mice exhibited a significant increase in hypertrophic chondrocytes and more severe cartilage damage post-DMM surgery compared to wild-type DMM mice (Figures 7A–D). To further assess the degree of cartilage damage, we performed Mankin’s scoring based on pathological staining results. Compared to the control group, the DMM mice had significantly higher Mankin scores, while Slit3 injection alleviated this change. However, Slit3^−/−^ mice showed higher scores than wild-type mice, with significantly higher scores post-DMM surgery (Figure 7E). Next, we analyzed the subchondral bone structure of the knee joints in 2-week group mice using micro-CT. The results showed that, compared to the control group, wild-type DMM mice had significantly reduced BV/TV and Tb.N, and significantly increased Tb.Th and Tb.Sp. Injection of Slit3 partially improved certain trabecular bone parameters; however, BV/TV was further decreased (Figures 7F–J). These experimental results confirm that Slit3 can inhibit early cartilage damage, degeneration, and structural changes in the subchondral bone of DMM mice.

We further evaluated the cartilage condition in 8-week group mice. The results revealed that, compared to the control group, wild-type DMM mice exhibited a loss of cartilage surface smoothness, an increase in hypertrophic chondrocytes, and a reduction in cartilage thickness. However, Slit3^−/−^ mice alleviated these changes (Supplementary Figures S10C–F). Mankin’s scores were consistent with the pathological staining results, showing that Slit3^−/−^ mice mitigated the significant increase in Mankin’s scores induced by DMM (Supplementary Figure S10G). These findings suggest that Slit3 contributes to late-stage cartilage degeneration progression in DMM mice. In conclusion, these experimental results indicate that Slit3 modulates structural changes in cartilage and subchondral bone, alleviating the early OA progression in DMM mice while exacerbating late-stage OA.

### 3.7 Slit3 promotes H-type angiogenesis and permeability in subchondral bone of DMM mice via Robo4/Rac1-GTP

**FIGURE 8:**
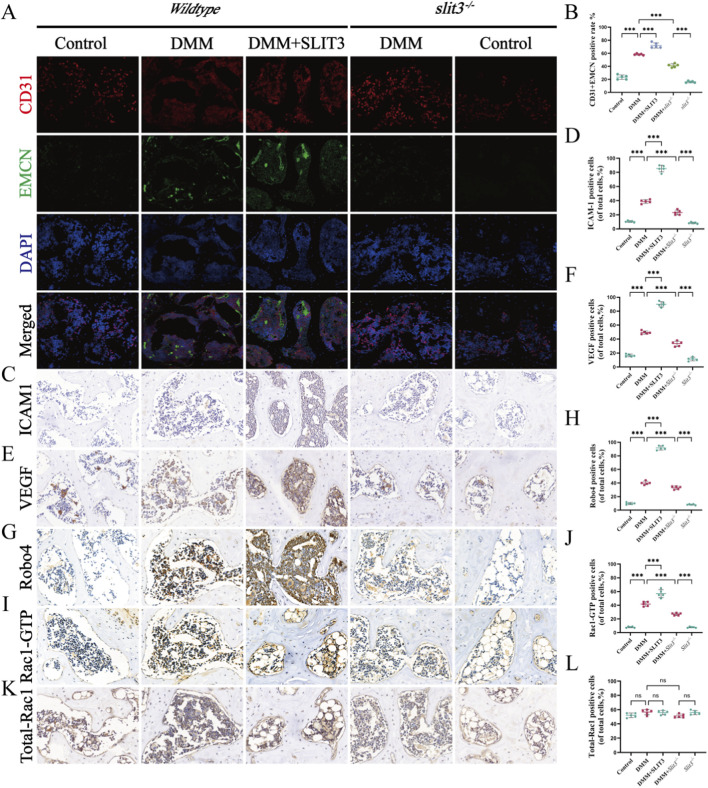
Slit3 promotes H-type angiogenesis and permeability in subchondral bone of DMM mice via Robo4/Rac1-GTP. **(A)** Representative immunofluorescence images of CD31 and EMCN in mouse knee joint tissues. Scale bar, 20 μm. **(B)** Quantification of the percentage of CD31+EMCN double-positive cells. n = 3. **(C)** Representative immunohistochemistry images of ICAM-1 in mouse knee joint tissues. Scale bar, 20 μm. **(D)** Quantification of the percentage of ICAM-1-positive cells. n = 3. **(E)** Representative immunohistochemistry images of VEGF in mouse knee joint tissues. Scale bar, 20 μm. **(F)** Quantification of the percentage of VEGF-positive cells. n = 3. **(G)** Representative immunohistochemistry images of Robo4 in mouse knee joint tissues. Scale bar, 20 μm. **(H)** Quantification of the percentage of Robo4-positive cells. n = 3. **(I)** Representative immunohistochemistry images of Total-Rac1 in mouse knee joint tissues. Scale bar, 20 μm. **(J)** Quantification of the percentage of Total-Rac1-positive cells. n = 3. **(K)** Representative immunohistochemistry images of Rac1-GTP in mouse knee joint tissues. Scale bar, 20 μm. **(L)** Quantification of the percentage of Rac1-GTP-positive cells. n = 3. All quantitative data are presented as mean ± standard deviation, *P < 0.05; **P < 0.01; ***P < 0.001. Comparisons between two groups were performed using Student’s t-test.

To further validate the role and mechanism of Slit3 in regulating H-type angiogenesis and permeability in the subchondral bone *in vivo*, we first performed immunofluorescence to detect biomarkers of H-type blood vessels (CD31^hi^EMCN^hi^). The results showed that, compared to the control group, the number of CD31+EMCN cells was significantly increased in the wild-type DMM group. Slit3 injection exacerbated this effect, while Slit3 knockout significantly suppressed the increase in CD31+EMCN cells in DMM mice (Figures 8A,B). Subsequently, we conducted immunohistochemistry to evaluate core molecules involved in angiogenesis and permeability. Compared to the control group, the wild-type DMM group showed a significant increase in ICAM-1 and VEGF-positive cells, which was further amplified by Slit3 injection. In contrast, Slit3 knockout inhibited the rise in ICAM-1 and VEGF-positive cells in DMM mice (Figures 8C–F). Lastly, we assessed the expression of Robo4, Rac1, and Rac1-GTP. The results indicated that the number of Robo4 and Rac1-GTP-positive cells was significantly elevated in the wild-type DMM group, and this increase was further aggravated by Slit3 injection. However, Slit3 knockout suppressed the increase in Robo4 and Rac1-GTP-positive cells induced by DMM (Figures 8G–J), whereas Rac1 expression showed no significant variation among these groups (Figures 8K,L). These findings are consistent with *in vitro* experiments and demonstrate that Slit3 promotes H-type angiogenesis and permeability in the subchondral bone of DMM mice through the Robo4/Rac1-GTP pathway.

## 4 Discussion

Although certain pathological changes in OA, such as cartilage degeneration and subchondral bone remodeling, have been well-established, the underlying molecular mechanisms remain unclear, which presents a significant challenge for timely OA treatment (Prieto-Alhambra et al., 2014). Therefore, identifying effective biomarkers and therapeutic targets for OA is of critical importance. The alteration of the subchondral bone microenvironment plays a pivotal role in the development and OA progression, involving bone remodeling, H-type angiogenesis, and nerve growth (Henrotin et al., 2012). Subchondral bone adapts dynamically to the mechanical stresses exerted on the joint through the coordination of bone remodeling, a process reliant on the coupling of osteoclast-mediated bone resorption and osteoblast-mediated bone formation, both of which are intimately associated with the generation and permeability of H-type blood vessels (Hu et al., 2021). H-type vessels are a newly identified capillary subtype associated with osteogenesis, characterized by high expression of CD31 and EMCN, and are found near the metaphyseal growth plate and the periosteum and endosteum (Kusumbe et al., 2014). Crosstalk between osteoblasts or osteoclast lineage cells and H-type ECs promotes subchondral angiogenesis and exacerbates subchondral bone remodeling (Percival and Richtsmeier, 2013; Bixel et al., 2024). H-type ECs, surrounding osteoblasts marked by osterix, secrete vascular endocrine factors such as PDGF-A and TGF-β1, which induce osteoblast proliferation and differentiation, thereby promoting local bone formation (Peng et al., 2020; Hasegawa et al., 2016). Moreover, TGF-β1 and other factors secreted by osteoblasts or osteoclast lineage cells act as pro-angiogenic factors, increasing the number of H-type ECs (Zhe et al., 2013; Zhao and Xie, 2020). In conclusion, H-type angiogenesis plays a crucial role in the alteration of the subchondral bone microenvironment in OA.

In this study, we identified Slit3 as a key regulator of H-type angiogenesis and vascular permeability in the subchondral bone during OA progression. While Slit3 is expressed in ECs under normal conditions, its expression is minimal. Upon inflammatory stimulation, Slit3 expression increases; however, its vascular-specific receptor, Robo4, does not show significant changes. Notably, Robo4 expression in the subchondral bone of patients with OA is significantly elevated, suggesting that the Robo4 receptor on ECs is not sensitive to inflammatory stimuli. Therefore, Slit3, which can bind to Robo4 in the subchondral bone microenvironment, may originate from sources other than ECs. Previous studies have shown that osteoblast-derived Slit3 increases the number of H-type ECs and promotes bone formation (Xu et al., 2018). Meanwhile, Slit3 from osteoclasts has been reported to stimulate bone formation while inhibiting bone resorption, exerting a protective effect on bone and serving as a potential biomarker for metabolic bone diseases (Kim et al., 2018). However, some studies argue that osteoclasts do not secrete Slit3 (Li et al., 2020). Overall, the main source of Slit3 in the subchondral bone microenvironment remains debated. To address this, our study used recombinant Slit3 protein to simulate Slit3 from non-EC sources in the subchondral bone environment. We found that Slit3 recombinant protein promoted the H-type EC phenotype (CD31+EMCN), and not only enhanced EC migration, invasion, and tube formation, but also reduced intercellular adhesion and increased actin filament formation—key factors for H-type vessel generation and permeability.

We further explored the mechanism underlying the action of Slit3 and discovered that Robo4 plays a key role in regulating H-type angiogenesis, which is consistent with our proteomic and clinical sample validation results. Robo4, a member of the Robo family, is distinct from other Robo family members (Robo1-3) that are primarily expressed in neurons of the central nervous system. Unlike these, Robo4 is specifically expressed in ECs, suggesting that Robo4 is involved in the physiological and pathological changes of ECs (Dickson and Gilestro, 2006; Blockus and Chédotal, 2016). However, the role of Slit3-Robo4 in regulating H-type angiogenesis in OA subchondral bone has not been studied. Our findings show that Robo4 neutralizing antibodies can inhibit the ability of Slit3 recombinant protein to promote EC migration, invasion, tube formation, and reduce intercellular adhesion. We further investigated the mechanism by which Robo4 affects H-type EC function and found that Robo4 neutralizing antibodies antagonized the Slit3-induced increase in Rac1-GTP and ROS. Active Rac1-GTP dynamically switches with inactive Rac1-GDP and is involved in regulating EC cytoskeleton, migration, proliferation, and differentiation. Our findings confirm that Rac1-GTP, rather than total Rac1, is involved in the Slit3-Robo4 regulation of H-type EC function, consistent with previous studies on Rac1 in ECs and our clinical sample results (Symons, 2011; Wang et al., 2016). We also found that inhibiting Rac1-GTP could counteract the Slit3-induced increase in H-type angiogenesis, vascular permeability, and ROS production. ROS, as a reactive oxygen species, plays multiple biological roles within cells, including involvement in signal transduction and regulation of gene expression (Cheung and Vousden, 2022). Studies have shown that inhibiting ROS in ECs can effectively reduce vascular permeability and maintain a low-inflammatory environment (Bielli et al., 2015). Furthermore, ROS can promote endothelial fibrosis, leading to downregulation of adhesion proteins, thereby disrupting endothelial integrity and increasing permeability (He et al., 2020; Jiang et al., 2020). Our study also revealed similar results, where inhibiting ROS counteracted the Slit3-induced increase in pro-angiogenic factor VEGF and actin filaments, rescuing EC permeability. Additionally, we observed that the effects of both Slit3 recombinant protein and Rac1-GTP inhibitors on EC permeability were time-dependent, with the strongest effects observed between 16 and 36 h and minimal effects after 48 h.

In in vivo experiments, knockout of the Slit3 gene was found to exacerbate cartilage degeneration and subchondral bone remodeling in DMM surgery mice at 2 weeks post-operation. This may be due to Slit3 promoting H-type angiogenesis and increased permeability, which enhances the local metabolic efficiency of the subchondral bone, providing necessary nutrients and oxygen for microfracture repair, reducing shear forces from microcracks on cartilage, and facilitating cartilage repair. These results align with previous studies showing that Slit3 promotes fracture healing (Xu et al., 2018). However, in DMM mice at 8 weeks post-operation, we observed that the knockout of Slit3 alleviated cartilage degeneration. This could be because, with the growth of H-type blood vessels and increased permeability in cartilage, abnormal vascularization contributes to increased cartilage calcification and degeneration. At this stage, the H-type blood vessels no longer provide protective effects for cartilage as they did in the early stages of OA. Additionally, we found that Slit3 can regulate the Robo4/Rac1-GTP axis *in vivo*.

**FIGURE 9:**
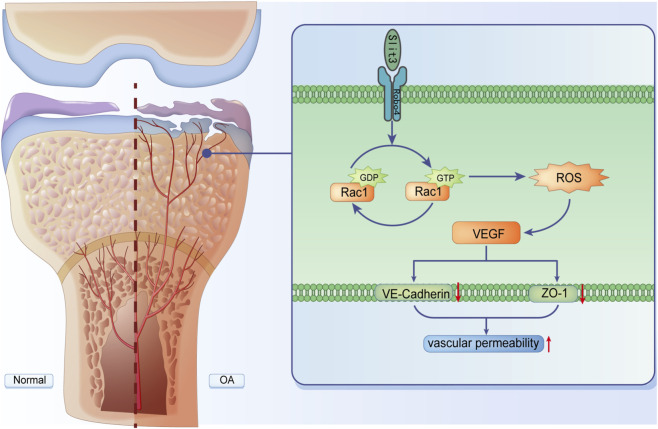
Slit3 promotes H-type angiogenesis and permeability in subchondral bone of OA via Robo4/Rac1-GTP/ROS.

In conclusion, our study reveals that Slit3 secreted by non-ECs in the subchondral bone microenvironment binds to Robo4 on ECs, which promotes Rac1-GTP activation without affecting Total-Rac1 and enhancing ROS production. This process induces EC migration, invasion, and tube formation; reduces EC adhesion; and increases actin filament formation, all of which contribute to the generation and permeability of H-type blood vessels. Through this mechanism, Slit3 alleviates early OA progression but exacerbates late-stage OA (Figure 9).

## Data Availability

The datasets presented in this study can be found in online repositories. The names of the repository/repositories and accession number(s) can be found below: https://proteomecentral.proteomexchange.org/ui?pxid=PXD051627.

## Supplementary material

SUPPLEMENTARY FIGURE S1 Histological sample data from patients with OA. **(A)** Representative weight-bearing anteroposterior X-ray, MRI imaging, and intraoperative joint cartilage images of three patients with knee OA. The severely damaged medial cartilage designated as the OA group (black box) and less damaged lateral cartilage designated as the control group (green box). **(B)** Preoperative OARSI score (0–20 points), visual analog scale (VAS) for pain (0–10 points), and K-L grade (0–4 points) of all patients. n = 30. **(C)** MRI-based measurement of cartilage thickness in the OA region (MF, MT) and control region (LF, LT) of the affected limb preoperatively. n = 30. **(D)** MRI-based measurement of subchondral bone thickness in the OA region (MT) and control region (LT) of the affected limb preoperatively. n = 30.

SUPPLEMENTARY FIGURE S2 Label-free quantitative proteomic analysis of knee joint subchondral bone tissue. **(A)** Heatmap of differentially expressed proteins. **(B)** Volcano plot of differentially expressed proteins. **(C-D)** GO and KEGG analysis results of differentially expressed proteins.

SUPPLEMENTARY FIGURE S3 Effects of different concentrations of **(A)** IL-1β and **(B)** NSC23766 on ECs viability.

SUPPLEMENTARY FIGURE S4 Selection of an appropriate concentration of Slit3 recombinant protein for ECs intervention. **(A)** Representative immunofluorescence images of slit3 in ECs after co-culturing with different concentrations of slit3 recombinant protein for 24 h. Scale bar, 50 μm. **(B)** Quantitative analysis of slit3 immunofluorescence intensity. **(C)** Quantitative analysis of the number of slit3 immunofluorescence-positive cells. All quantitative data are presented as mean ± standard deviation, *P < 0.05; **P < 0.01; ***P < 0.001. A comparison between two groups was performed using the Student's t-test.

SUPPLEMENTARY FIGURE S5 Selection of an appropriate concentration of Slit3 neutralizing antibody for ECs intervention. Endothelial cells were co-treated with IL-1β (100 ng/mL) and varying concentrations of Slit3 neutralizing antibody (0, 0.5, 1, 1.25and 1.5 μg/mL) for 24 h. **(A)** Representative immunofluorescence images of slit3 in ECs. Scale bar, 50 μm. **(B)** Quantitative analysis of slit3 immunofluorescence intensity. **(C)** Cell viability was determined using the CCK-8 assay. All quantitative data are presented as mean ± standard deviation, *P < 0.05; **P < 0.01; ***P < 0.001. A comparison between two groups was performed using the Student's t-test.

SUPPLEMENTARY FIGURE S6 Exogenous addition of slit3 promotes permeability of H-type vascular ECs. ECs were co-cultured with exogenous slit3 recombinant protein (1.25 μg/mL) or slit3 neutralizing antibody (1.25 μg/mL) for 24 h. **(A)** Representative immunofluorescence images of ZO-1. Scale bar, 50 μm. **(B)** Quantification of average ZO-1 fluorescence intensity. n = 3. **(C)** Representative immunofluorescence images of Connexin-43. Scale bar, 50 μm. **(D)** Quantification of average Connexin-43 fluorescence intensity. n = 3. **(E)** Representative immunofluorescence images of VE-Cadherin. Scale bar, 20 μm. **(F)** Representative immunofluorescence images of ECs actin cytoskeleton. Scale bar, 20 μm. All quantitative data are presented as mean ± standard deviation, *P < 0.05; **P < 0.01; ***P < 0.001. A comparison between two groups was performed using the Student's t-test.

SUPPLEMENTARY FIGURE S7 Robo4 neutralizing antibody inhibits slit3-induced increases in EC migration, invasion, tube formation ability, and ROS activation. ECs were co-cultured with exogenous Slit3 recombinant protein (1.25 μg/mL) alone or together with Robo4 neutralizing antibody (1.25 μg/mL) for 24 h. **(A)** Representative crystal violet-ammonium oxalate staining images of ECs migrating through a 3 μm pore membrane. Scale bar, 100 μm. **(B)** Quantification of the number of migrated ECs. n = 3. **(C)** Representative crystal violet-ammonium oxalate staining images of ECs invading through a matrix-gel covered 3 μm pore membrane. Scale bar, 100 μm. **(D)** Quantification of the number of invading ECs. n = 3. **(E)** Representative images of ECs forming tubes on matrix-gel. Scale bar, 50 μm. **(F)** Quantification of the number of tube-like structures per field of view. **(G)** Representative immunofluorescence images of ROS. Scale bar, 50 μm. **(H)** Quantification of ROS immunofluorescence intensity. n = 3. All quantitative data are presented as mean ± standard deviation, *P < 0.05; **P < 0.01; ***P < 0.001. Comparisons between two groups were performed using the Student’s t-test.

SUPPLEMENTARY FIGURE S8 Rac1-GTP inhibition suppresses slit3-induced increase in H-type ECs phenotype. **(A)** Representative immunofluorescence staining images of CD31+EMCN. Scale bar, 50 μm. **(B)** Quantification of the percentage of CD31+EMCN positive cells. n = 3. All quantitative data are presented as mean ± standard deviation, *P < 0.05; **P < 0.01; ***P < 0.001. Comparisons between two groups were performed using the Student’s t-test.

SUPPLEMENTARY FIGURE S9 Slit3 promotes H-type vascular formation and permeability through ROS/Rac1-GTP. **(A)** Representative immunofluorescence staining images of ROS after co-culturing ECs with slit3 recombinant protein, NSC23766, NAC, or slit3 with NSC23766 or NAC for 24 h. Scale bar, 50 μm. **(B)** Quantification of ROS immunofluorescence intensity. n = 3. **(C)** Representative immunofluorescence staining images of VEGF after co-culturing ECs with slit3 or NAC, or slit3 with NAC for 24 h. Scale bar, 50 μm. **(D)** Quantification of VEGF-positive cell count by immunofluorescence. n = 3. **(E)** Representative immunofluorescence staining images of ECs actin cytoskeleton. Scale bar, 20 μm. **(F)** Quantification of actin cytoskeleton staining. n = 3. All quantitative data are presented as mean ± standard deviation, *P < 0.05; **P < 0.01; ***P < 0.001. Comparisons between two groups were performed using the Student’s t-test.

SUPPLEMENTARY FIGURE S10 Knockout of Slit3 alleviates advanced (8-week-old) DMM mouse OA by regulating cartilage and subchondral bone structure. **(A)** Representative immunohistochemistry images of Slit3 in mouse knee joint tissue. Scale bar, 20 μm. **(B)** Quantification of Slit3 immunohistochemistry-positive cell proportion. n = 3. **(C)** Representative H&E staining images of mouse knee joint tissue. Scale bar, 100 and 50 μm. **(D)** Representative Safranin O-fast green staining images of mouse knee joint tissue. Scale bar, 100 and 50 μm. **(E)** Representative Masson staining images of mouse knee joint tissue. Scale bar, 100 and 50 μm. **(F)** Quantification of knee joint cartilage thickness. n = 3. **(G)** Mankin’s scoring for cartilage pathology. n = 3. **(H)** Immunoblot analysis of Aggrecan and MMP13. **(I,J)** Quantification of Aggrecan and MMP13 protein expression. n = 3. All quantitative data are presented as mean ± standard deviation, *P < 0.05; **P < 0.01; ***P < 0.001. Comparisons between two groups were performed using the Student’s t-test.
