# Supplementary figures and images for: Regulation of H-type angiogenesis and permeability in the subchondral bone of osteoarthritis: the role of Slit3 and the Robo4/Rac1-GTP/ROS axis
Source: Front Cell Dev Biol. 2026 May 1;14:1787805. doi: 10.3389/fcell.2026.1787805 (PMC13176150; doi:10.3389/fcell.2026.1787805)

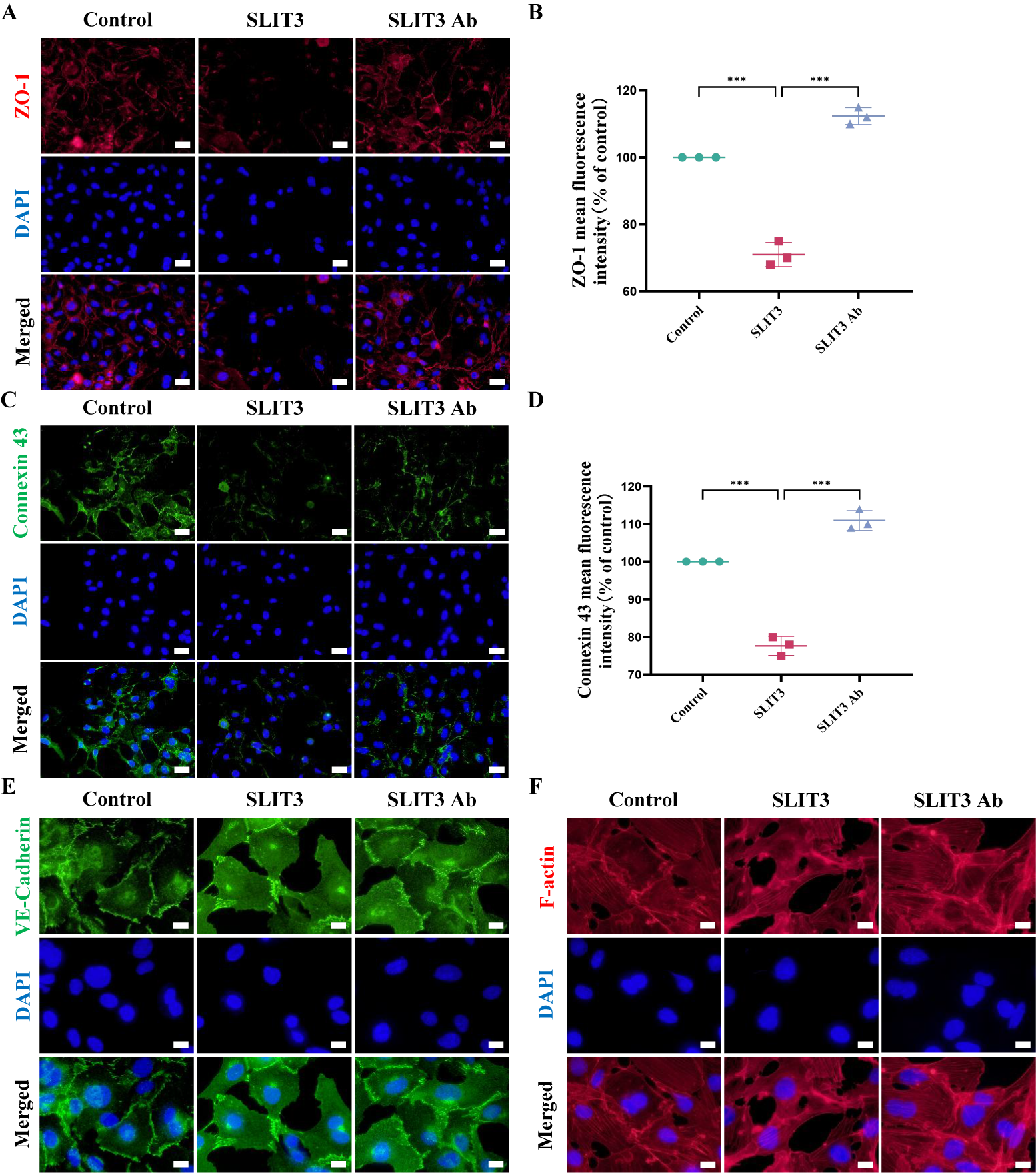

Supplement: Supplementary file 1 [file Image6.tif]

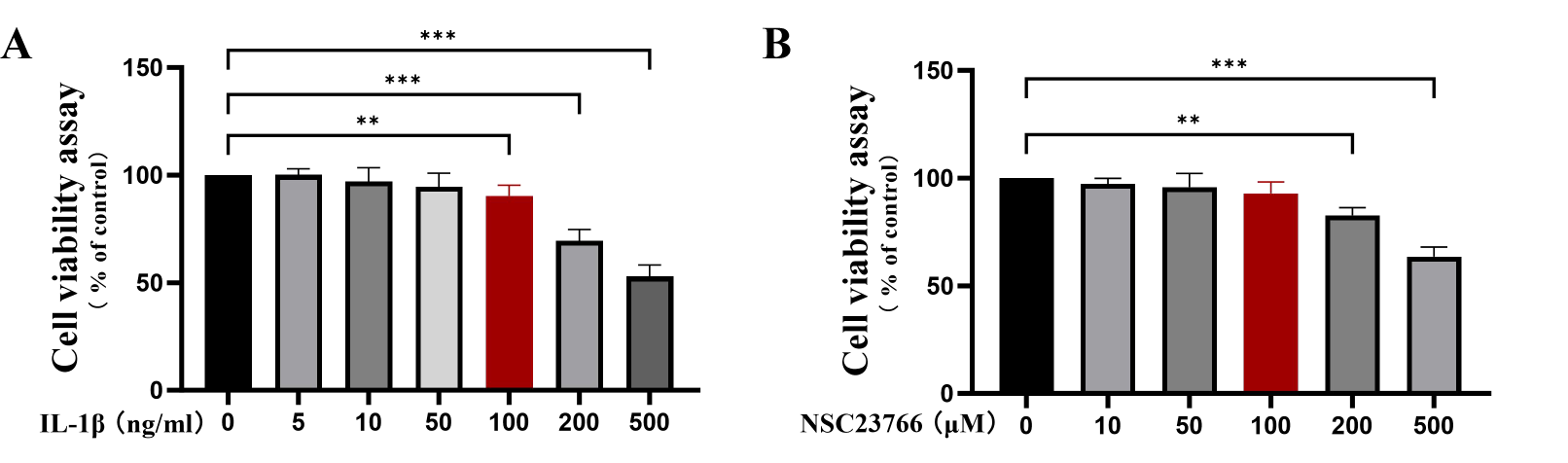

Supplement: Supplementary file 2 [file Image3.tif]

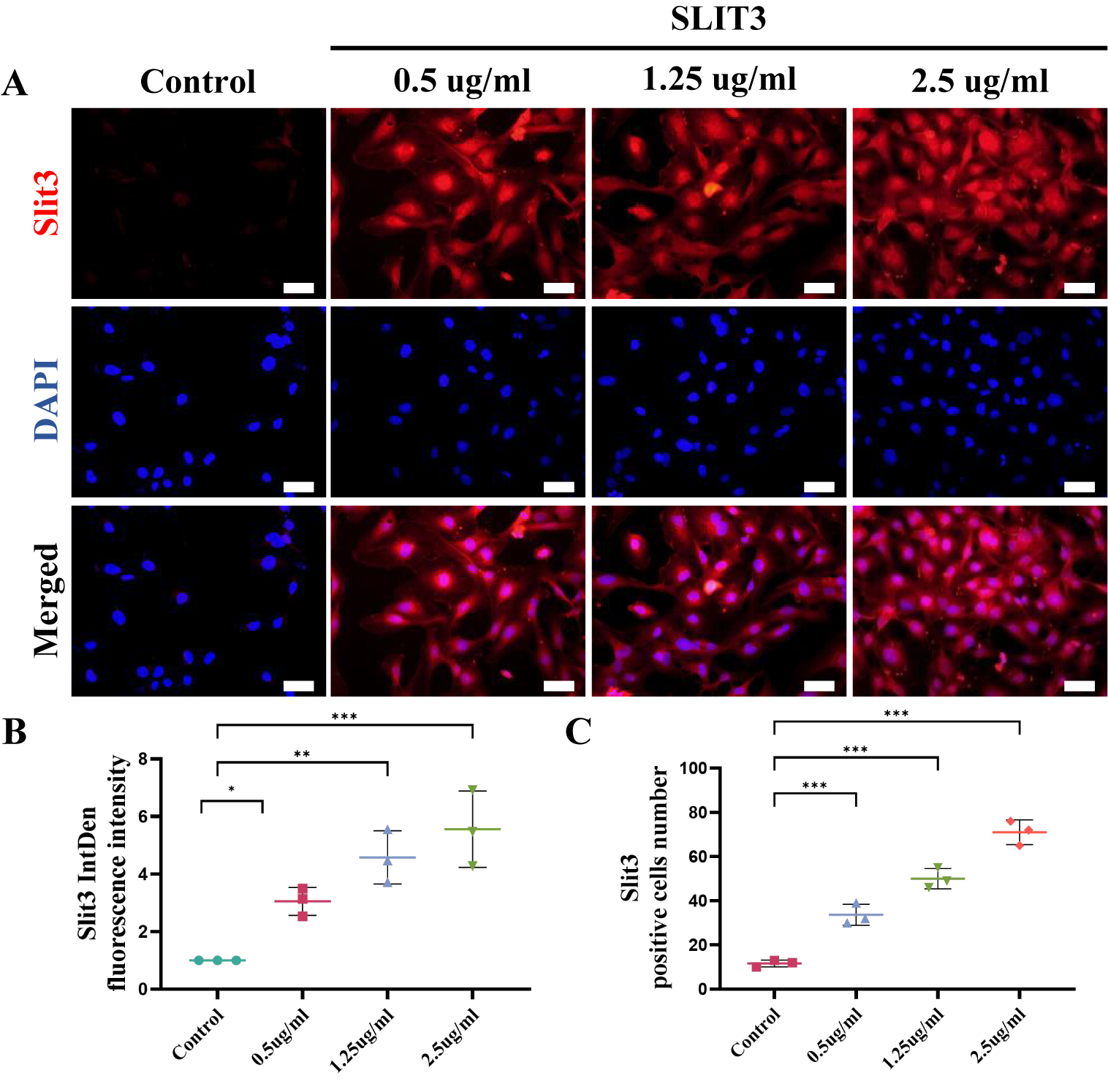

Supplement: Supplementary file 3 [file Image4.tif]

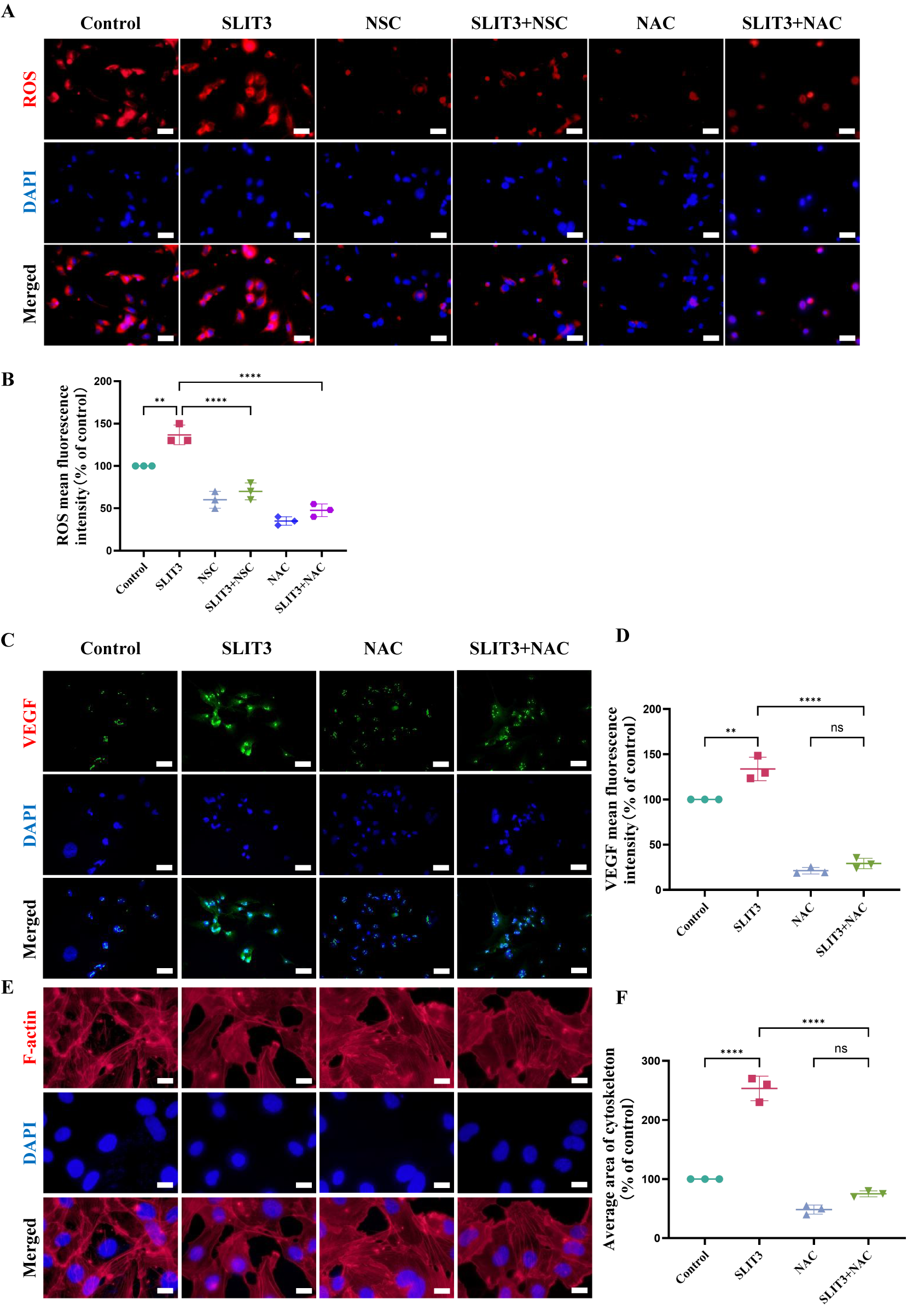

Supplement: Supplementary file 4 [file Image9.tif]

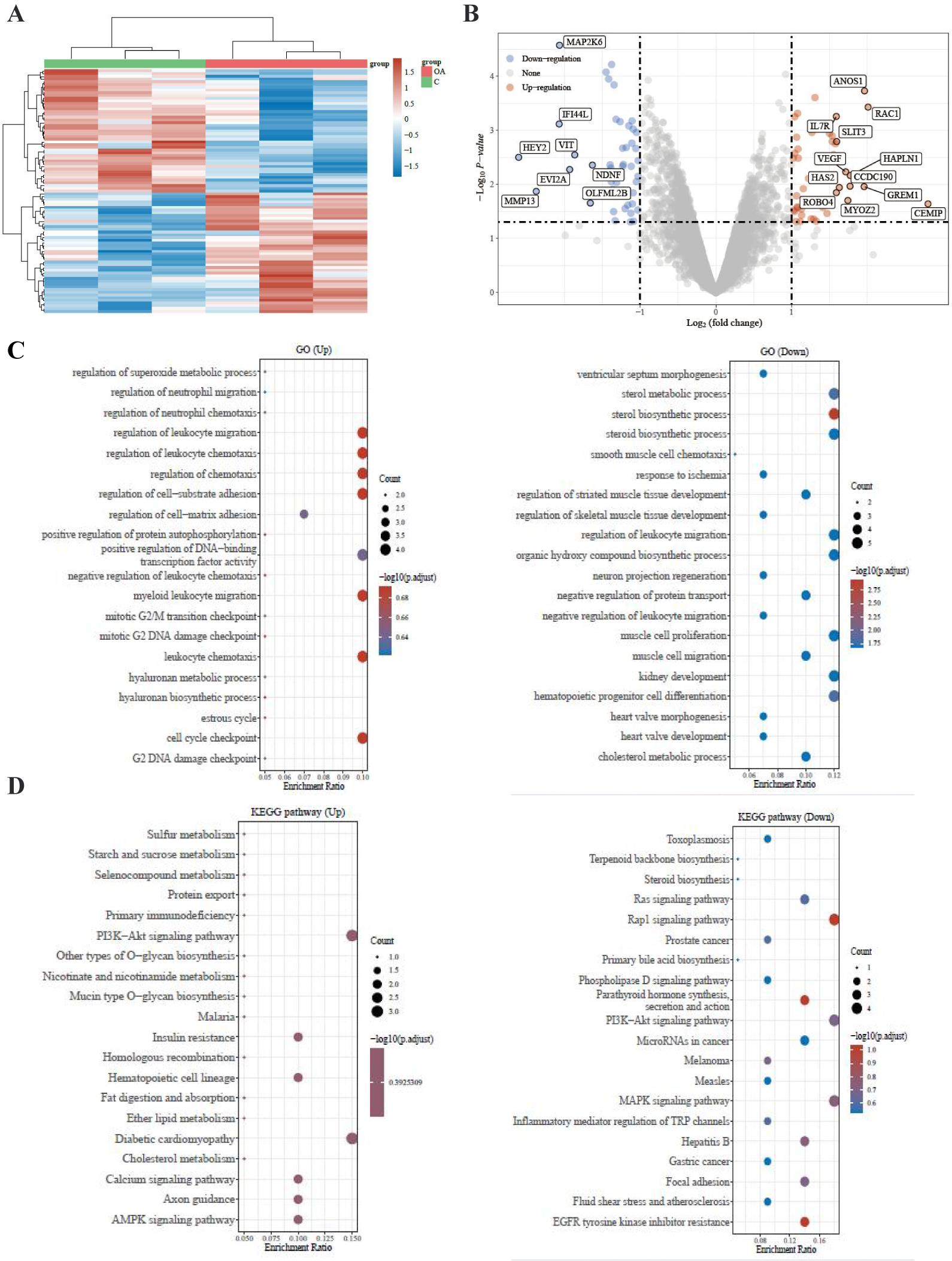

Supplement: Supplementary file 5 [file Image2.tif]

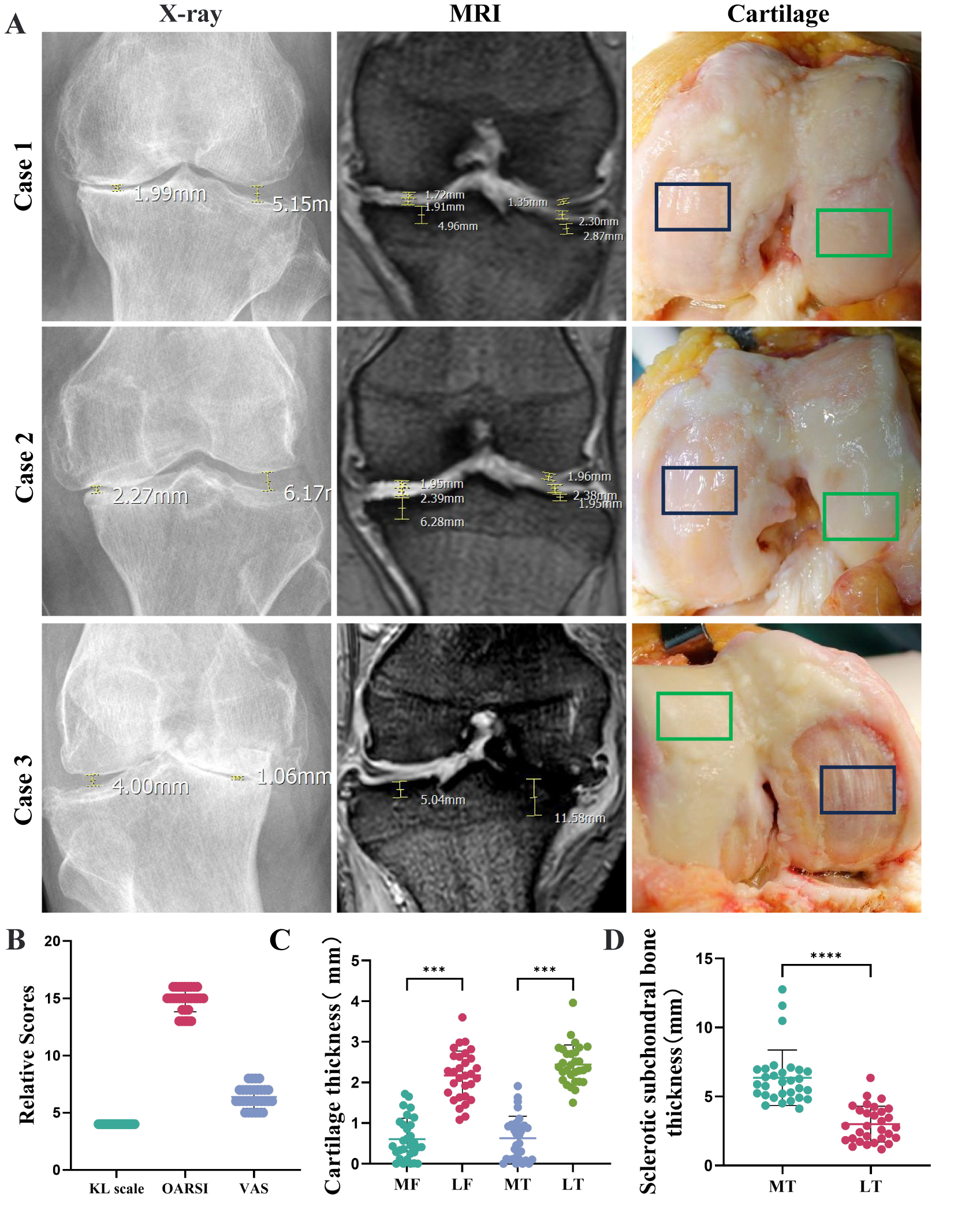

Supplement: Supplementary file 6 [file Image1.tif]

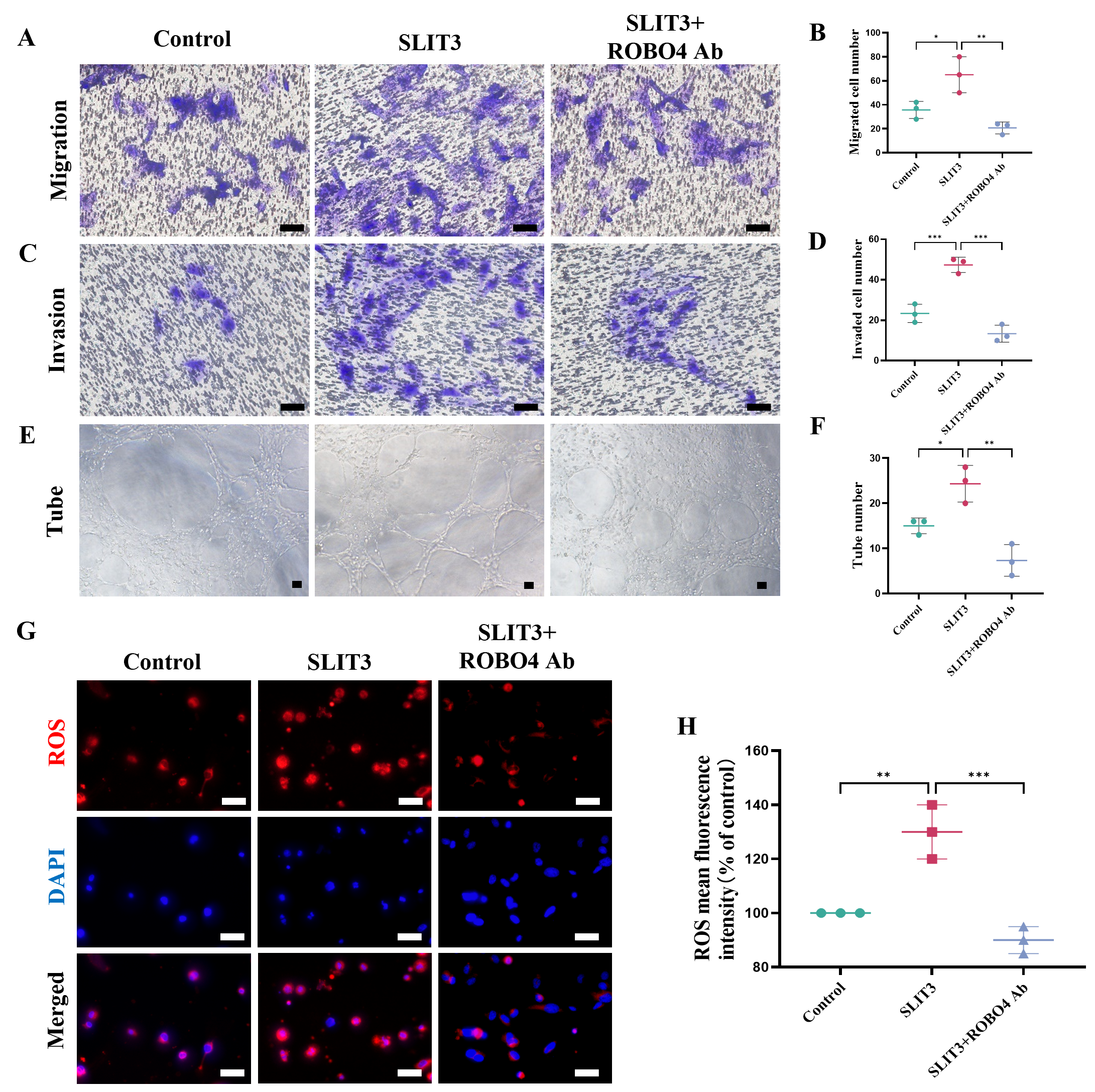

Supplement: Supplementary file 7 [file Image7.tif]

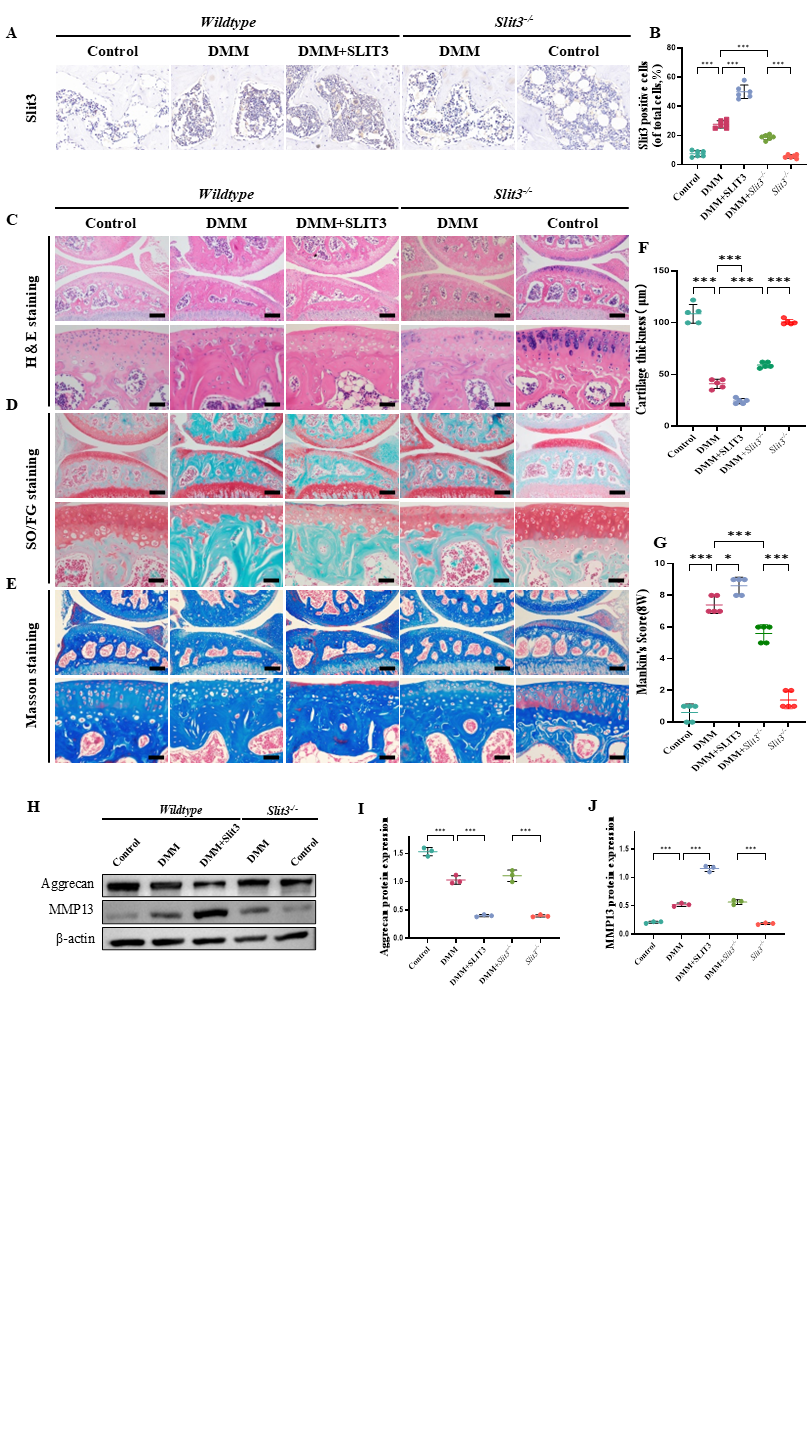

Supplement: Supplementary file 8 [file Image10.tiff]

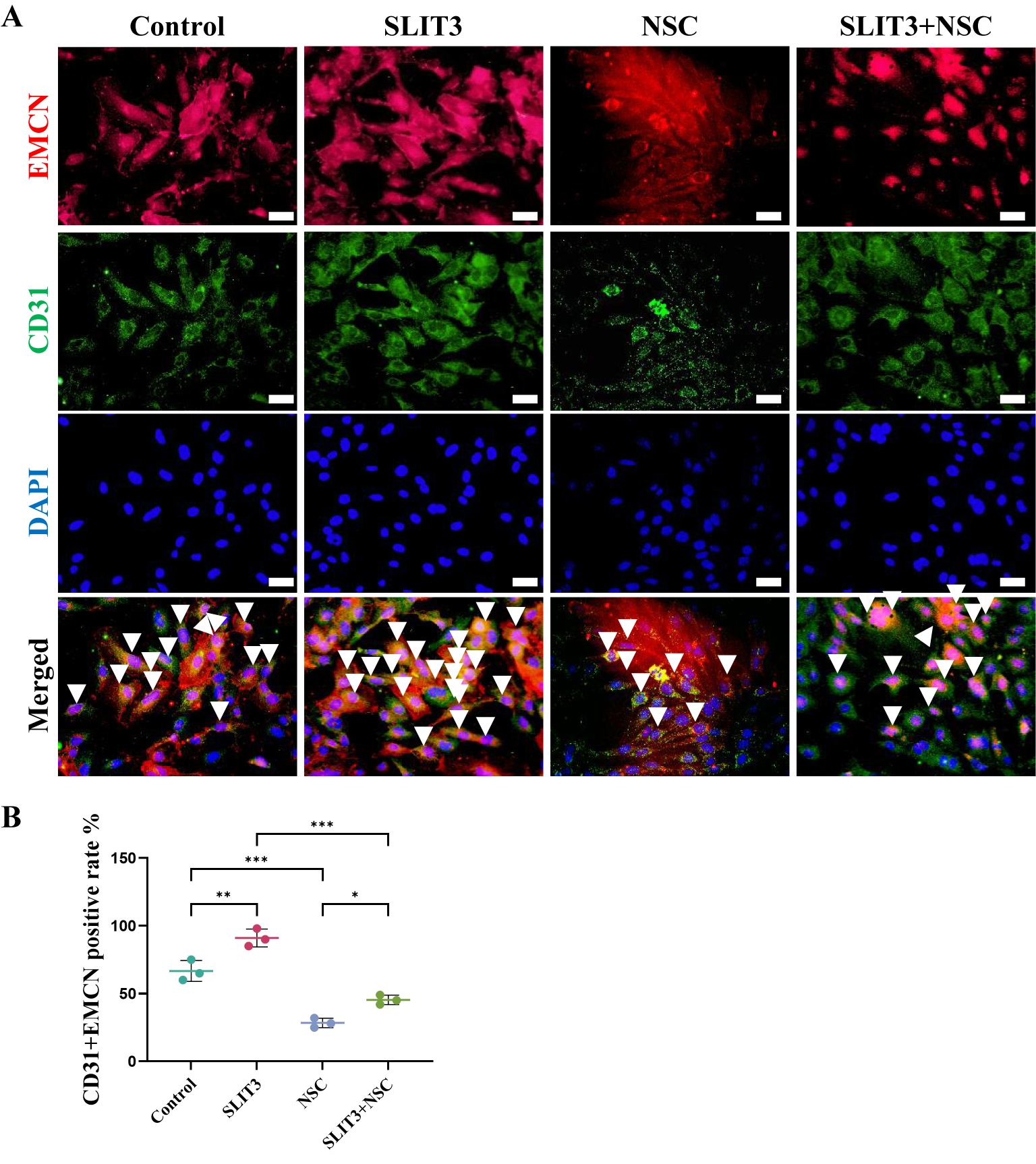

Supplement: Supplementary file 9 [file Image8.tif]

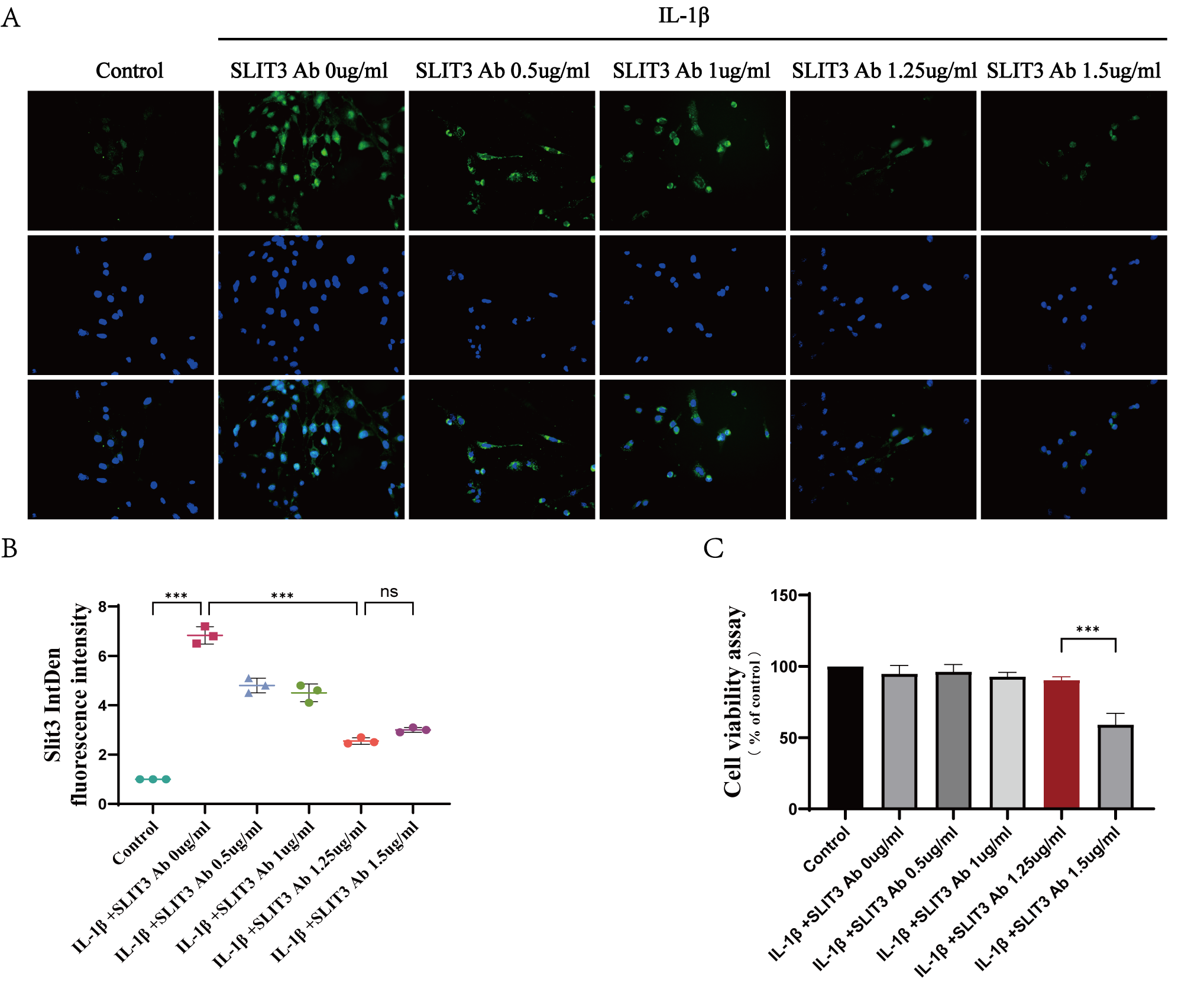

Supplement: Supplementary file 10 [file Image5.tif]
